# Experimental and Theoretical Study of Stable and Metastable Phases in Sputtered CuInS_2_

**DOI:** 10.1002/advs.202200848

**Published:** 2022-06-20

**Authors:** Jes K. Larsen, Kostiantyn V. Sopiha, Clas Persson, Charlotte Platzer‐Björkman, Marika Edoff

**Affiliations:** ^1^ Division of Solar Cell Technology Department of Materials Science and Engineering Uppsala University Box 534 Uppsala SE‐75237 Sweden; ^2^ Centre for Materials Science and Nanotechnology/Department of Physics University of Oslo Blindern, Box 1048 Oslo NO‐0316 Norway; ^3^ Department of Materials Science and Engineering Royal Institute of Technology Stockholm SE‐10044 Sweden

**Keywords:** disorder, polymorphs, polytypes, Raman spectra, wurtzite, zinc‐blende

## Abstract

The chalcopyrite Cu(In,Ga)S_2_ has gained renewed interest in recent years due to the potential application in tandem solar cells. In this contribution, a combined theoretical and experimental approach is applied to investigate stable and metastable phases forming in CuInS_2_ (CIS) thin films. Ab initio calculations are performed to obtain formation energies, X‐ray diffraction (XRD) patterns, and Raman spectra of CIS polytypes and related compounds. Multiple CIS structures with zinc‐blende and wurtzite‐derived lattices are identified and their XRD/Raman patterns are shown to contain overlapping features, which could lead to misidentification. Thin films with compositions from Cu‐rich to Cu‐poor are synthesized via a two‐step approach based on sputtering from binary targets followed by high‐temperature sulfurization. It is discovered that several CIS polymorphs are formed when growing the material with this approach. In the Cu‐poor material, wurtzite CIS is observed for the first time in sputtered thin films along with chalcopyrite CIS and CuAu‐ordered CIS. Once the wurtzite CIS phase has formed, it is difficult to convert into the stable chalcopyrite polymorph. CuIn_5_S_8_ and NaInS_2_ accommodating In‐excess are found alongside the CIS polymorphs. It is argued that the metastable polymorphs are stabilized by off‐stoichiometry of the precursors, hence tight composition control is required.

## Graphical abstract

A combined theoretical and experimental approach is applied to investigate stable and metastable phases of CuInS_2_ (CIS). Multiple CIS polytypes are identified and analyzed by ab initio calculations. Thin films are deposited via sputtering followed by sulfurization, yielding a mixture of CIS polymorphs and secondary phases. A wurtzite polymorph is found particularly difficult to convert into the stable chalcopyrite phase.

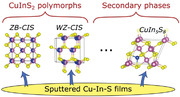

## 1 Introduction

The current world record for power conversion efficiency of Cu(In,Ga)(Se,S)_2_‐based solar cells was obtained by utilizing a “sulfurization‐after‐selenization” process.^[^ ^1^ ^]^ This is done to incorporate sulfur in the surface of the absorber and widen the band gap near the junction with the buffer layer. The resulting record efficiency of 23.35% is not far behind the world record for crystalline Si solar cells, which is currently 26.6%—only a few percentage points below the theoretical limit of 29.1% for Si.^[^ ^2^ ^]^ An established way of increasing solar cell efficiency beyond the current level is by making tandem or multi‐junction devices. For a four‐terminal tandem with a silicon bottom cell, it is theoretically possible to achieve efficiencies around 44% using a top cell with a band gap in the range 1.6−2.0 eV.^[^ ^3^ ^]^ One candidate material for top cells in combination with Cu(In,Ga)Se_2_ or Si is the sulfide Cu(In,Ga)S_2_. Its band gap can be tuned from 1.5 eV for CuInS_2_ to 2.4 eV for CuGaS_2_, which covers the desired range for top cell applications. Historically, sulfide Cu(In,Ga)S_2_ materials have received less attention than the more established selenide Cu(In,Ga)Se_2_ because they are less suited for the traditional single‐junction photovoltaics. The interest in the sulfides is, however, increasing with the enhanced focus on tandem solar cells. Early research has primarily focused on Cu‐rich material,^[^ ^4^ ^]^ because conductivity was always too low for Cu‐poor films,^[^ ^5^, ^6^ ^]^ but after initial advances the record efficiency for Cu‐rich Cu(In,Ga)S_2_ had saturated at around 13% despite extensive academic and industrial efforts.^[^ ^7^ ^]^ Recently, a breakthrough was reported for Cu(In,Ga)S_2_ solar cells reaching a conversion efficiency of 15.5% using Cu‐poor material,^[^ ^8^ ^]^ which raises questions about the best processing route. This was further supported in a recent publication demonstrating 15.2% efficiency for a Cu(In,Ga)S_2_‐based device by reducing the [Cu]/([Ga]+[In]) ratio and utilizing a Zn(O,S) buffer layer to suppress interface recombination.^[^ ^9^ ^]^ Motivated by the improved efficiency, we are set to revise the details of growth chemistry in Cu‐poor CuInS_2_ (CIS). Besides solar cells, the obtained results might also be of interest for thermoelectric applications^[^ ^10^, ^11^ ^]^ and beyond.

**Figure 1:**
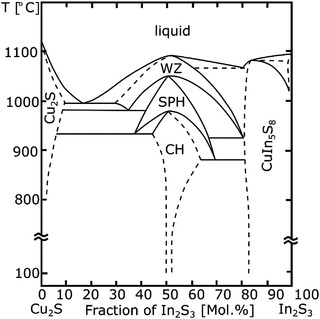
Pseudo‐binary phase diagram of Cu_2_S‐In_2_S_3_ system. Adapted with permission.^[^ ^12^ ^]^ Copyright 2022, Elsevier.

The first complete pseudo‐binary phase diagram of the Cu_2_S‐In_2_S_3_ system was published in 1980 by Binsma et al.^[^ ^12^ ^]^ (see **Figure** **1**). It shows that CuInS_2_ exists in the chalcopyrite phase with a tetragonal structure (CH‐CIS; space group *I* $\bar{4}$ *2d*) at room temperature and transforms into disordered zinc‐blende‐like sphalerite phase (SPH‐CIS; space group *F* $\bar{4}$ *3m*) at 980 °C, followed by conversion into disordered wurtzite‐like phase (WZ‐CIS; space group *P6_3_mc*) at 1045 °C. Both disordered phases are characterized by random occupation of cationic sites while the anionic sublattice is filled by S atoms. Unlike many I‐III‐VI_2_ isomorphs, chalcopyrite CuInS_2_ has surprisingly narrow homogeneity region—only a few percent toward the In_2_S_3_‐rich side. Thiospinel CuIn_5_S_8_ and Cu_2_S form at the In‐rich and Cu‐rich compositions, respectively. Interestingly, the phase diagram also shows that slightly off‐stoichiometric CIS remain disordered at up to 100 °C lower temperature compared to stoichiometric CuInS_2_, suggesting that SPH‐CIS and WZ‐CIS phases can be stabilized by either excess or deficiency of Cu_2_S. A similar stabilization effect was noted in selenide Cu‐In‐Se system, where the SPH phase could be observed down to room temperature when the composition fell between 15.5 and 11.0 at% Cu on the CuInSe_2_‐In_0.42_Se_0.58_ tie‐line (considerably Cu‐poor with respect to chalcopyrite CuInSe_2_ and Se‐depleted as compared to the ordered vacancy compound CuIn_3_Se_5_‐like phase).^[^ ^13^ ^]^ All these findings highlight the intricate interplay between stoichiometry and stability of CuInS_2_ polymorphs, which needs to be duly accounted for during the deposition of solar absorbers.

The renewed interest in Cu(In,Ga)S_2_ absorbers stimulated a detailed study of the crystalline phases observed in the Cu_2_S‐In_2_S_3_‐Ga_2_S_3_ pseudo‐ternary system in 2018.^[^ ^14^ ^]^ In that study, Thomere et al. point out that sulfide Cu(In,Ga)S_2_ is much more structurally complex than its selenide counterpart. It is particularly important that the sulfide chalcopyrite structure is less adaptable to Cu‐deficiency, reflecting the narrow homogeneity range reported by Binsma et al.^[^ ^12^ ^]^ They concluded that any attempt to produce Cu‐poor Cu(In,Ga)S_2_ in sealed silica tubes at 800 °C resulted in the formation of mixed chalcopyrite and Cu‐poor phases such as the thiospinel CuIn_5_S_8_. A few years earlier, C. Stephan investigated the extension of the single‐phase region of CIS and observed CuIn_5_S_8_ precipitates together with CH‐CIS already for [Cu]/[In] < 0.95, while CH‐CIS and Cu*_x_* S were found to co‐exist for [Cu]/[In] > 1,^[^ ^15^ ^]^ also in accordance with the phase diagram.^[^ ^12^ ^]^ It should be noted that these studies were performed on powder samples produced under nearly equilibrium conditions (prolonged annealing at 800 °C or above in sealed quartz tubes followed by slow cooling^[^ ^14^, ^15^ ^]^), with the composition being intentionally set on the Cu_2_S‐In_2_S_3_ tie‐line by controlling the elemental precursor ratios. In contrast, solar absorber films are typically deposited under the conditions far from equilibrium, with S deficiency or excess being possible during the growth, thereby opening the possibility to form metastable phases.

**Figure 2:**
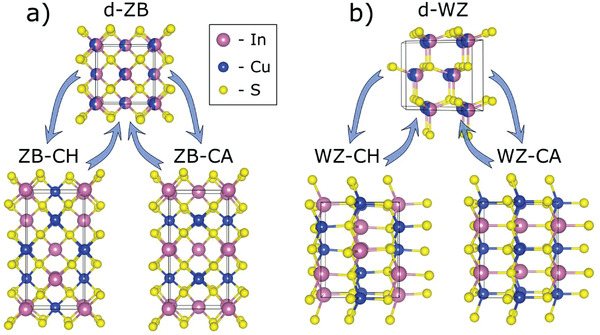
Structures of the a) ZB‐ and b) WZ‐based CuInS_2_ phases discussed in this work. The structures labeled “d‐” are the disordered phases. The CH‐like and CA‐like structures are obtained through cationic ordering, as indicated with the arrows.

Apart from the disordered SPH‐CIS and WZ‐CIS, and the ordered ground state CH‐CIS phase, three other, potentially metastable ordered structures have been described. These are a CuAu‐type structure based on a zinc‐blende lattice (ZB‐CA; space group *P* $\bar{4}$ *m2*), as well as two structures based on the wurtzite lattice with chalcopyrite‐type (WZ‐CH; space group *Pna2_1_*) and CuAu‐type ordering (WZ‐CA; space group *Pmc2_1_*).^[^ ^10^, ^16^ ^]^ **Figure** **2** illustrates how the disordered phases transform into either the CH‐like or CA‐like structure upon ordering. All four ordered structures obey Grimm‐Sommerfeld rule,^[^ ^17^ ^]^ often referred to as octet rule, which is a known prerequisite of low energy structures.^[^ ^18^, ^19^ ^]^ In principle, infinite number of polytypes can be derived without violating the octet rule by, for example, incorporating antisite domain boundaries into CH‐CIS^[^ ^20^, ^21^ ^]^ or choosing a different mixing pattern for the wurtzite lattice.^[^ ^11^ ^]^ Many of those structures were reproduced and analyzed computationally in this work.

It is important to differentiate between “phases” and “structures” as these terms do not necessarily refer to the same thing. For instance, disordered phases can often be represented as statistical ensembles of ordered structural polytypes, or as structures with fractional site occupancies. The crystal structures of the CH‐CIS, CA‐CIS, and SPH‐CIS phases are known to be ZB‐CH, ZB‐CA, and d‐ZB, respectively. Thus, these notations are equivalent and are thus used when referring to either structures or phases in this paper. The structure of the WZ‐CIS phase, in contrast, has not yet been fully resolved, necessitating distinctions between WZ‐CH, WZ‐CA, and d‐WZ.

**Figure 3:**
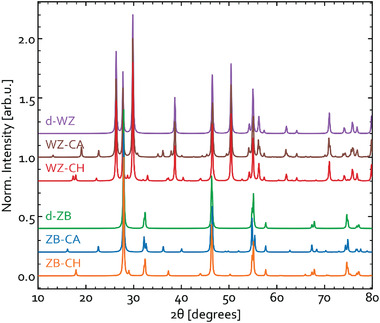
XRD patterns for different CIS structures (under Cu K*α* radiation). The patterns were simulated using XRD diffraction module in pymatgen, which is based on the formalism laid out elsewhere.^[^ ^31^ ^]^ To add spectral broadening, Lorentzian smearing with FWHM of 0.2^°^ was applied to all patters presented.

With such a diversity of structures, one should not be surprised of the lacking consensus in literature about the types and properties of metastable CIS phases. The main practical problem is that, owing to the structural similarity, all ZB‐derived structures produce overlapping diffraction peaks, making it difficult to unambiguously identify them by X‐ray diffraction (XRD). CH‐CIS has only a couple of unique reflections, most notably the (011) peak at 2*θ* ≈ 17.9° when using Cu K*α* radiation,^[^ ^22^ ^]^ whereas CA‐CIS has unique (001) reflection at 2*θ* ≈ 16.1° and (100) reflection at 2*θ* ≈ 22.6°, both of which are not seen in the other CIS polymorphs.^[^ ^23^, ^24^, ^25^ ^]^ For reference, the respective diffraction patterns are given in **Figure** **3**. In principle, these features should be enough to detect CH‐CIS or CA‐CIS in samples containing SPH‐CIS but insufficient to rule out the presence of SPH‐CIS in a mixture with CH‐CIS or CA‐CIS. Moreover, in practice, the unique XRD reflections for ZB‐derived phases are rarely strong enough to detect in thin films or small nanocrystals, rendering them unreliable for phase identification. Still, XRD has been adopted as the primary tool to conclude that polycrystalline powder produced via high‐pressure solid‐state reaction^[^ ^23^ ^]^ or epitaxially grown films via molecular beam epitaxy on silicon (001) substrate^[^ ^24^ ^]^ can be composed predominantly of the CA‐CIS polymorph. It should be noted though that there is also a number of reports suggesting that epitaxial^[^ ^25^, ^26^, ^27^, ^28^ ^]^ and co‐evaporated^[^ ^29^, ^30^ ^]^ films can consist of mixed CH‐CIS and CA‐CIS. Another notable observation in literature is that the fraction of CA‐CIS phase increases when lowering the [Cu]/[In] ratio in co‐evaporated films,^[^ ^29^, ^30^ ^]^ suggesting that it can tolerate higher off‐stoichiometry than CH‐CIS.

Different WZ‐derived structures produce similar diffraction patterns but all distinct from those of ZB‐derived CIS (see Figure 3). This was first noted experimentally in 1998 by Schimmel et al.,^[^ ^32^ ^]^ who detected WZ‐CIS in electrodeposited CIS nanoparticles by XRD. However, it was not until 2008 when Pan et al.^[^ ^33^ ^]^ discovered a simple way to toggle between ZB‐ and WZ‐derived phases by changing the capping agent in hot‐injection synthesis that WZ‐CIS started to be reliably produced, primarily via low‐temperature (below 300 °C) wet‐chemical routes.^[^ ^11^, ^22^, ^34^, ^35^, ^36^, ^37^, ^38^ ^]^ Another parameter that was claimed to trigger preferential growth of WZ‐CIS is the solutions pH.^[^ ^37^ ^]^ Such phase tuning indicates that WZ‐CIS is stabilized by lowering surface energy in the solution during growth. This effect must be particularly important for nanocrystals due to their high specific surface area.^[^ ^10^ ^]^ At the same time, the smaller contribution of surface energy in thin films justifies why no earlier study has detected WZ‐CIS precipitation in sputtered CIS films.

A handful of experimental evidence exists that the cationic disorder vanishes at room temperature. First, Binsma et al.^[^ ^12^ ^]^ noted that freezing SPH‐CIS and WZ‐CIS by quenching in liquid N_2_ is impossible. Instead, using Monte‐Carlo simulations based on the energies of CuInSe_2_ structures computed using the density functional theory (DFT), Wei et al.^[^ ^18^ ^]^ concluded that disordered I‐III‐VI_2_ at room temperature attain mixed CH‐ and CA‐type features if the material is grown below the critical order‐disorder transition temperature. Subsequently, the presence of CA‐CIS domains in epitaxially grown CIS film was detected with high‐resolution transmission electron microscopy^[^ ^26^, ^27^, ^39^ ^]^ shortly thereafter. Likewise, Shen et al.^[^ ^11^ ^]^ found that WZ‐CIS nanocrystals grown by a solution‐phase colloidal synthesis are composed of interlaced domains of ordered wurtzite structures. This conclusion was later confirmed elsewhere.^[^ ^40^ ^]^

Raman spectroscopy is more effective than XRD when it comes to detection of CA‐CIS. It was proven that CA‐CIS has a A_1_‐S‐S vibration mode at about 5% higher wavenumbers than CH‐CIS, appearing at about 305 cm^−1^ for CA‐CIS as compared to 292 cm^−1^ for CH‐CIS.^[^ ^39^ ^]^ At the same time, untangling Raman signatures for the WZ‐CIS phases is ambiguous—a few benchmark studies exist,^[^ ^41^, ^42^ ^]^ but they all show several peaks that may overlap with the A_1_‐S‐S modes of ZB‐derived CIS and/or other secondary phases, such as CuIn_5_S_8_. Dzhagan et al. suggested that the key identifier of WZ‐CIS is a peak at 340–350 cm^−1^, which is found to be sensitive to the choice of excitation wavelength.^[^ ^41^ ^]^ An alternative explanation is that this signal stems from B_2_^(3)^(L)/E^(6)^(L) mode of CH‐CIS experiencing quasi‐resonance enhancement under excitation by infrared laser (*λ*_exc_ = 785 nm).^[^ ^43^, ^44^ ^]^ Another complication for phase identification is that, to our best knowledge, neither computed nor measured reference Raman spectra exists for disordered SPH‐CIS and WZ‐CIS in literature.

In this work, a joint experimental and theoretical approach is applied in order to enhance understanding of metastable CIS polymorphs. Ab initio DFT calculations are performed to obtain formation energies, XRD patterns, and Raman spectra of CIS polymorphs and related In‐rich phases. The simulation results are compared to measurements performed on CIS samples produced via a two‐step CIS fabrication process that relies on 1) compound sputtering of a Cu‐In‐S precursor and 2) subsequent annealing in a sulfur‐containing atmosphere. A similar approach to CIS preparation has been explored in the past (see for example refs. [5, 45]), while an alternative one‐step sputtering route is likewise commonly employed (e.g., refs. [46, 47]). The produced films are thoroughly characterized and found to contain varying amounts of CIS phases depending on the precursor composition and processing conditions. Based on this data, crystal structures and growth tendencies for metastable CIS phases are assessed and discussed.

## 2 Experimental Section

### 2.1 Sample Preparation

#### 2.1.1 Sputtering and Annealing of Cu‐In‐S

Prior to CIS deposition, soda‐lime glass (SLG) substrates were coated with about 350 nm Mo by DC‐magnetron sputtering. A series of Cu‐In‐S precursors were deposited onto Mo by co‐sputtering from In_2_S_3_ and CuS binary targets without intentional substrate heating. Samples with different [Cu]/[In] ratios were prepared by variation of the relative power of the targets and the resulting compositions of the precursors were measured by X‐ray fluorescence (XRF). For some samples, 20 nm NaF was deposited onto the precursors by evaporation before annealing. The precursors were subsequently annealed at various temperatures in a tube furnace described in more details in ref. [48]. In preparation for the annealing, the precursors were loaded in a pyrolytic carbon coated graphite box containing 180 mg of elemental sulfur. The graphite box was then introduced into the preheated furnace with an Ar background pressure of 350 Torr. After the samples were transferred into the hot zone, the box was heated up to the target temperature in about 90 s. The samples were then allowed to dwell for 30 min before the graphite box was removed from the hot zone, resulting in a rapid cool down in Ar atmosphere. The reported sample temperature was measured at the base of the graphite box. No sulfur remained in the box after annealing.

#### 2.1.2 Reference Material Preparation

A NaInS_2_ thin‐film reference sample was produced in order to determine the characteristic Raman peaks of the material. The sample was grown on soda‐lime glass by first depositing 50 nm of NaF using evaporation, followed by thermal evaporation of 30 nm of metallic indium. The resulting precursor is estimated to have [Na]/[In] close to 1.5, ensuring substantial Na excess. To sulfurize the material, the precursor sample was loaded into a graphite carrier with 20 g of elemental sulfur and introduced into a custom‐built furnace described in more details in ref. [49]. The graphite carrier containing the sample was moved into the preheated graphite reactor and annealed at 580 °C for 30 min in an Ar atmosphere with a background pressure of 300 mbar. In Figure S1, Supporting Information, it is demonstrated that the XRD pattern of the NaInS_2_ layer matches the database pattern for the compound with the *R‐3m* space group.^[^ ^50^ ^]^ The Raman spectra of the NaInS_2_ sample measured with 532, 633, and 785 nm excitation wavelengths are available in Figure S2, Supporting Information.

A CuIn_5_S_8_ thin‐film reference sample was prepared with an approach similar to NaInS_2_. Cu and In were deposited on a Mo‐coated high‐strain glass (with low Na content) by co‐evaporation. The composition of the precursor was verified by XRF to be [Cu]/[In] = 0.2. This metallic precursor was then sulfurized following the same procedure as described for NaInS_2_ above. Figure S3, Supporting Information shows the XRD pattern of this sample, and it appears to be single‐phase CuIn_5_S_8_. Figure S4, Supporting Information shows the multi‐wavelength Raman spectra of the sample.

An In_2_S_3_ thin‐film reference sample was prepared by annealing a pellet of metallic indium in sulfur following the same procedure as described above for the NaInS_2_ sample.

### 2.2 Materials Characterization

Scanning electron microscopy (SEM) was performed in a Zeiss LEO 1550 microscope. An acceleration voltage of 10 kV was used for all measurements. Raman spectroscopy was carried out at room temperature in a Renishaw inVia system using lasers with wavelengths of 532, 633, and 785 nm. Power densities in the range of 5−50 W cm^−2^ were used. Grazing incidence (GI) and Bragg‐Brentano XRD were performed with a Siemens D5000 system using Cu K*α* radiation. All GIXRD measurements were performed with an incidence angle (*d*_inc_) of 1°.

### 2.3 First‐Principles Calculations

#### 2.3.1 Computational Setup

The first‐principles calculations within DFT were carried out using the Vienna Ab initio Simulation Package (VASP)^[^ ^87^ ^]^ employing projector augmented wave method^[^ ^88^ ^]^ and Perdew–Burke–Ernzerhof (PBE)^[^ ^89^ ^]^ exchange‐correlation functional. Pseudopotentials with the valence electron configurations of Cu 3d^10^4s^1^, In 5s^2^5p^1^, S 3s^2^3p^4^, Na 3s^1^, and the cut‐off energy of 350 eV were used. For the formation energy calculations, relatively dense Γ‐centered Monkhorst–Pack grids^[^ ^90^ ^]^ with about 8000 k‐points per reciprocal atom were adopted. Both lattice parameters and ionic positions were optimized when calculating formation energies until all ionic forces decreased below 10 meV Å^−1^. All calculations were performed in the non‐spin‐polarized mode. The parameters used in the calculations for the Raman spectra simulations, when different from the already specified, are presented in Section 2.3.6.

#### 2.3.2 Data Processing and Visualization

Throughout the study, preparation of input files and analysis of results were greatly facilitated by the use of pymatgen (Python Materials Genomics) library,^[^ ^51^ ^]^ whereas the 3D visualization of structures was done using the Visualization for Electronic and Structural Analysis (VESTA) software.^[^ ^52^ ^]^

#### 2.3.3 Polytype Generation Algorithm

To generate CuInS_2_ polytypes, a recursive algorithm was developed for filling all cationic sites in various supercells of the primitive zinc‐blende (two atoms) or wurtzite (four atoms) cells with either Cu or In to reach the intended [Cu]/[In] = 1. All supercells containing up to 48 atoms for ZB‐derived and 64 atoms for WZ‐derived structures were considered. The starting set of inequivalent supercells was generated using the Alloy Theoretic Automated Toolkit (ATAT)^[^ ^53^ ^]^ and then filling of the cationic sites was performed by a custom python script. As the goal was to produce structures obeying the octet rule, on‐the‐fly check function was incorporated to filter out all structures violating it. Moreover, the structures were constantly compared to the already identified ones and removed on‐the‐fly whenever duplication was discovered. There was no beforehand requirement to produce polytypes consisting of mixed CH‐ and CA‐type domains only—such a motif was discovered later upon inspection of the generated dataset. The resulting dataset of structures after the ionic relaxation is accessible from the Materials Cloud repository^[^ ^54^ ^]^ under the following identifier.^[^ ^55^ ^]^

#### 2.3.4 Assignment of CA‐type Fractions

The CA‐type fraction was ascribed to every polytype based on the fraction of Cu atoms that have the same local coordination (within the second coordination sphere, i.e., with the nearest cations) as in the ordered CuInS_2_ prototypes (i.e., ZB‐CH, ZB‐CA, WZ‐CH, or WZ‐CA).

#### 2.3.5 Simulation of XRD Patterns

The XRD patterns were simulated using diffraction module in pymatgen,^[^ ^51^ ^]^ which is based on the formalism laid out elsewhere.^[^ ^31^ ^]^ X‐ray wavelength of 1.5406 Å corresponding to Cu K*α* was assumed. To add spectral broadening, Lorentzian smearing with the full width at half maximum (FWHM) of 0.2° was applied to all patters.

#### 2.3.6 Simulation of Raman Spectra

The off‐resonance Raman spectra were simulated using “vasp_raman.py” utility written by Fonari and Stauffer,^[^ ^56^ ^]^ which in operation relies on the formalism described by Porezag and Pederson.^[^ ^57^ ^]^ The method requires two ingredients: 1) frequencies of phonons at Γ‐point and 2) derivatives of macroscopic dielectric tensor with respect to atomic displacements for each mode. Both of these were computed within the density functional perturbation theory,^[^ ^58^ ^]^ as implemented in VASP package. The derivatives of dielectric tensor were computed numerically by displacing the atoms 0.01 Å in both positive and negative directions. Only the modes with wavenumbers above 100 cm^−1^ were analyzed. Instead of the PBE functional used for the formation energy analysis, PBE+U functional with the Hubbard U correction of 5 eV applied on Cu 3d was adopted for these calculations. The U correction was applied according to the formalism developed by Dudarev et al.^[^ ^59^ ^]^ The change in functional was necessitated by severe underestimation of the band gap energy by PBE, yielding inadequate Raman spectra. The magnitude of U correction energy was chosen based on the previous computational studies of CuInSe_2_,^[^ ^60^, ^61^, ^62^ ^]^ and it was found to deliver the intended band gap opening (although the band gap of 0.43 eV computed for CH‐CIS after lattice relaxation was still grossly underestimated). Other Hubbard U values were tested and found to produce similar spectra. Prior to the phonon calculations, all structures were transformed into cells containing at least 14 atoms. To eliminate residual forces, atomic positions were re‐optimized using a stricter atomic force threshold of 1 meV Å^−1^. Based on our test, we found that experimental vibration frequencies are better reproduced when the lattice constants are fixed to their experimental values. As such, experimental lattices from refs. [50, 63, 64, 65, 66] were set for secondary phases. For all ZB‐ and WZ‐derived structures, the lattices of CH‐CIS^[^ ^67^ ^]^ and WZ‐CIS^[^ ^68^ ^]^ were used as references to scale the optimized cell vectors by a constant such that per‐atom volumes of ZB‐CH and WZ‐CH cells matched the respective experimental values. The partially disordered CuIn_5_S_8_ (space group *F* $\bar{4}$ *3m*)^[^ ^64^ ^]^ was converted into its ordered analogue by filling all mixed‐cation sites by Cu and In in a checkerboard order. The k‐point density was changed to about 2500 points per reciprocal atom. For ordered CIS and secondary phases, the cut‐off energy was set to 550 eV, whereas for polytypes this parameter remained equal 350 eV (the difference in Raman spectra was found to be negligible). The raw data files and optimized structures can be accessed under the following identifier.^[^ ^55^ ^]^ To account for the spectral line broadening, all modes delivered by the vasp_raman.py script were smeared by the Lorentz function with FWHM of 10 cm^−1^, unless otherwise specified.

### 2.4 Statistical Analysis and Raw Data

The statistical analysis performed in the computational part of this study was limited to the construction of average XRD and Raman spectra for a collection of structures (ensembles) to describe cation‐disordered CIS phases, as described below. The raw data and graphs for individual structures are accessible from the Materials Cloud archive.^[^ ^55^ ^]^ The only pre‐processing performed on the experimental data was normalization of all XRD and Raman measurements.

## 3 Results

### 3.1 Ab Initio Analysis of CuInS_2_ Polymorphs: Structures and Energies

The possibility of forming different CuInS_2_ polymorphs depending on the growth method is typically attributed to the small difference in their formation energies. This consideration is most often applied to ZB‐CA, the energy of which is only about 2 meV atom^−1^ higher than of the ground state ZB‐CH.^[^ ^19^, ^28^ ^]^ Wurtzite CuInS_2_ has also been found unstable with respect to ZB‐CH, but the reported energies are surprisingly inconsistent.^[^ ^41^, ^69^ ^]^ Still, Shen et al.^[^ ^11^ ^]^ identified that WZ‐CH has 1 meV atom^−1^ lower energy than WZ‐CA. This agrees well with our calculations showing that metastable ZB‐CA, WZ‐CH, and WZ‐CA have respectively 1.6, 5.4, and 6.2 meV atom^−1^ higher energy than the ground state ZB‐CH.

The formation energies of disordered d‐ZB and d‐WZ phases are harder to assess. Experimentally, these are described as ideal lattices with partial cationic site occupancies. Computationally, the disorder is replicated by randomly filling the cationic sublattice with Cu and In in a sufficiently large supercell, yielding structures with large deviation from the octet rule. Several earlier reports have shown that presence of such deviations—(3In+Cu) and (3Cu+In) tetrahedra in the statistical mix—increases energy by 0.3–0.4 eV per unit.^[^ ^11^, ^18^ ^]^ Due to the high energy cost, random distribution of cations should only be expected at high temperatures (we refer to it as the “high‐temperature” model of disorder) and transform into a more stable mixture of ordered phases upon cooling.^[^ ^18^ ^]^ When the disordered phases are grown at room or moderate temperature through colloidal synthesis, co‐existing domains of ordered structures with zero deviation from the octet rule (and thus lower energy) are thought to form instead, as it was described for WZ‐CIS nanoparticles before.^[^ ^11^ ^]^ This scenario should be better represented by an ensemble of ordered low‐energy polytypes with zero deviation from the octet rule. This type of disorder is referred to as the “room‐temperature” model herein.

**Figure 4:**
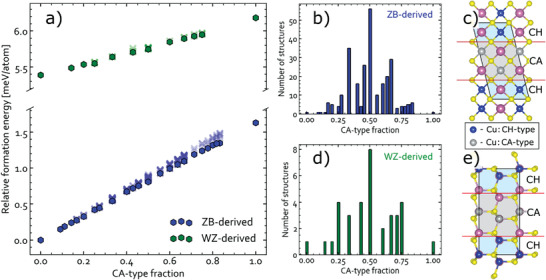
Ground‐state stability of CuInS_2_ polytypes. a) Formation energy of different CuInS_2_ polytypes with respect to that of ZB‐CH versus the CA‐type fraction. The hexagons represent the most stable structures for a given CA‐type fraction; all other polytypes are represented by crosses. Distribution of b) ZB‐ and d) WZ‐derived polytypes by the CA‐type fraction. Examples of low‐energy c) ZB‐ and e) WZ‐derived polytypes with 50% CA‐type fraction. Other polytypes can be accessed via the following identifier.^[^ ^55^ ^]^

The computed energies and two representative polytypes are depicted in **Figure** **4**. This figure compiles data for 269 ZB‐based and 35 WZ‐based polytypes, and it reveals linear correlations between the CuAu‐type fraction and formation energies for both lattices. No dependence on the cell size was found and very little energy variation is seen for polytypes with the same CuAu‐type fraction, meaning that they have the same probability of appearing in the material. This result means that, provided the stacking order does not violate the octet rule, interfacial energy between different polytypes is essentially zero. This result echoes the argumentation presented by Shen et al. for the interlaced WZ‐CIS nanoparticles^[^ ^11^ ^]^ and suggests that ZB‐based CIS nanoparticles might have a similar compound morphology.

**Figure 5:**
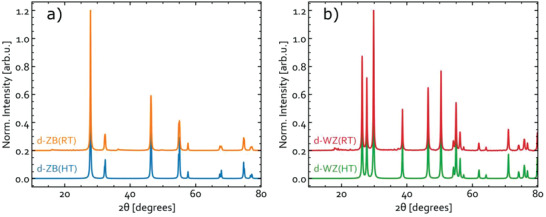
Simulated XRD patterns for the fully cation‐disordered (as seen at high temperature; HT) and ensemble‐averaged (as expected at room‐temperature; RT) representations of a) d‐ZB and b) d‐WZ phases. The d‐ZB(RT) and d‐WZ(RT) patterns were obtained by averaging the simulated patterns for 269 ZB‐based and 35 WZ‐based polytypes, whereas the d‐ZB(HT) and d‐WZ(HT) patterns are simulated by ascribing each cationic site the same partial occupancy of 50% In and 50% Cu. For all structures, the DFT‐optimized geometries were scaled to match per atom volume of the experimental CH‐CIS^[^ ^67^ ^]^ or WZ‐CIS structures.^[^ ^68^ ^]^

To demonstrate the impact of such compound morphology on the material characterization, we first simulated and then averaged XRD patterns for all polytypes with ZB‐ and WZ‐derived structures. The results can therefore be treated as features of the metastable disordered phases of CIS (within the room‐temperature model). We find that this approach is similar to that developed by Jones and Stevanović for glassy solids,^[^ ^70^ ^]^ but more primitive as we ascribe all the structures identical statistical weights, which we think is a reasonable approximation for the room‐temperature disordered material. The obtained patterns are shown alongside those for the ordered and high‐temperature disordered phases in **Figure** **5**. As one can see, all unique reflections seen for the ordered phases vanish in the polytype‐averaged patterns but resemble those for the high‐temperature disordered ZB‐CIS and WZ‐CIS. This similarity illustrates that the high‐ and room‐temperature disordered materials are indistinguishable with XRD, explaining why no distinction has been conceived in experimental studies.

It is also interesting to point out that, accepting the linear correlation between formation energy and CA‐type fraction, both ZB‐CA and WZ‐CA must be unstable at all temperatures. This is because the configuration entropy, which contributes greatly to free energy at elevated temperatures, is higher for macrostates with a large number of microstates, peaking at 50% CA‐type fraction for CIS, as hinted by Figure 4b,d. Such macrostates should therefore stabilize upon heating. Since ZB‐CA and WZ‐CA are ordered, their configuration entropies are zero. As a result, any kinetic trapping necessary for the formation of metastable CA‐type phases must be impossible. At the first glance, this conclusion contradicts numerous experimental observations, but the caveat is that ZB‐CA in experiment has been characterized for samples produced by methods that can tip the energy balance in favor of ZB‐CA (e.g., possible substrate strains in epitaxial films,^[^ ^24^, ^25^, ^26^, ^27^, ^28^ ^]^ pressure–volume contributions to free energy for high‐pressure synthesis^[^ ^23^ ^]^). At the same time, for the films grown by co‐evaporation, the increased yield of ZB‐CA in Cu‐poor CIS might reflect the stabilization by off‐stoichiometry.^[^ ^29^, ^30^ ^]^ The discrepancy might also be rooted in the effects or parameters overlooked by our calculations (e.g., vibration entropies). Herein, we do not attempt to identify the root cause but simply highlight the discrepancy for future studies.

### 3.2 Identification of Secondary Phases in Cu‐Poor CuInS_2_ by Raman Spectroscopy Using Reference Materials and Ab Initio Calculations

Raman spectroscopy is a powerful technique that allows identification of individual phases in a phase mixture with micrometer resolution. It has, for example, been successful in distinguishing phases in Cu_2_ZnSnS_4_‐based materials that are not identifiable by XRD.^[^ ^71^ ^]^ It can be useful to recognize contributions from various phases in CuInS_2_‐based absorbers, too. It is known that CIS grown under Cu‐rich conditions results in the formation of stoichiometric CIS and Cu*_x_* S.^[^ ^12^ ^]^ Since the Raman spectrum of Cu*_x_* S is well known (main peak at 475 cm^−1[^ ^72^ ^]^), we herein focus on the phases that can form along with CIS when grown with a Cu‐deficiency. The three phases found in the Cu‐poor part of the phase diagram are CuInS_2_, CuIn_5_S_8_, and In_2_S_3_. Even though In_2_S_3_ is not expected in any of the samples based on their stoichiometry, we include it here since the samples are grown under non‐equilibrium conditions. In addition to the phases expected from the phase diagram, NaInS_2_ was found to form due to the Na supply from the soda‐lime glass. NaInS_2_ has previously been observed in Cu‐poor CIS prepared via reactive sputtering followed by annealing in H_2_S,^[^ ^5^ ^]^ and found to have Raman signatures at 158 and 289 cm^−1^.^[^ ^73^ ^]^ The Raman spectrum of CuIn_5_S_8_ has also been documented in literature. The spectrum measured with an excitation wavelength of 514 nm was reported in 1991 by Gasanly et al.^[^ ^74^ ^]^ This data formed the basis for identification of CuIn_5_S_8_ in a phase mixture with CIS in several subsequent studies.^[^ ^34^, ^75^ ^]^ The Raman spectrum of phase‐pure CuIn_5_S_8_ has, however, not been reported with other excitation wavelengths, and is therefore worth examining. The Raman signature of In_2_S_3_ is better documented in literature (e.g., efs. [76, 77, 78]). The spectra of In_2_S_3_ measured in this work are presented in Figure S5, Supporting Information.

**Figure 6:**
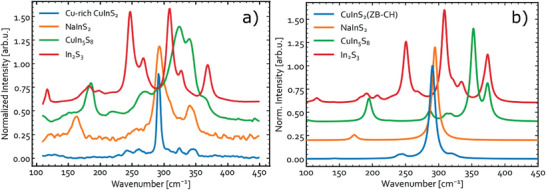
Raman spectra of stable phases in the CuInS_2_‐In_2_S_3_ pseudo‐binary system. a) Experimental spectra recorded using 633 nm excitation laser. The NaInS_2_ film is measured with 5× higher excitation density than the other samples due to much weaker signal. b) Simulated off‐resonance Raman spectra for the same compounds.

The Raman spectra of all reference materials measured with an excitation wavelength of 633 nm are compiled in **Figure** **6**a. The spectra measured with 532 and 785 nm excitation are included in Figures S6 and S7, Supporting Information.

Included in Figure 6a is the Raman spectrum of Cu‐rich CIS ([Cu]/[In] = 1.39). It is presented here in order to compare the reference samples to CIS free of phases that form under Cu‐poor conditions. It is immediately clear that In_2_S_3_ is easily distinguishable from the other phases. The spectrum is dominated by peaks at 117, 138, 180, **247**, 267, **308**, 327, and 368 cm^−1^ (bold font style indicates most prominent peaks), in good agreement with previous reports for *β*‐In_2_S_3_.^[^ ^76^, ^77^, ^78^ ^]^ CuIn_5_S_8_ has peaks at **186**, 267, **324**, **340**, and 360 cm^−1^, which is in agreement with the paper by Gasanly et al.,^[^ ^74^ ^]^ who observed a similar spectrum with relatively broad features and the most prominent peaks at 327, 341, and 360 cm^−1^. The peak at 186 cm^−1^ was less pronounced in the spectrum by Gasanly et al. This is likewise the case in our study when using the 532 nm excitation, as seen in Figure S4, Supporting Information. The Raman spectrum of NaInS_2_ is characterized by three main peaks at 163, **293**, and 341 cm^−1^, while the CuInS_2_ sample has peaks at 243, 260, **292**, 324, and 345 cm^−1^, with the 292 cm^−1^ mode clearly dominating the spectrum. This spectrum is typical for Cu‐rich CIS measured under non‐resonant conditions.^[^ ^75^ ^]^ As one notices, the main mode of NaInS_2_ is overlapping with the dominant signal for CH‐CIS, which in the latter case is attributed to A_1_ vibration mode of S—S bonds.^[^ ^79^ ^]^ Thus, the 293 cm^−1^ mode in NaInS_2_ may have the same origin. It is noticed that the peaks in NaInS_2_ are significantly wider than in CIS. This may be an indication of poorer crystal quality due to the non‐optimized synthesis of NaInS_2_. Based on the spectra presented in Figure 6a, it is clear that almost all the phases have unique peaks and may be distinguishable by Raman spectroscopy given that a large fraction of the phase is present in the probed volume. The main difficulty lies in the overlap of the main modes of CH‐CIS and NaInS_2_. Furthermore, the Raman response of NaInS_2_ was found to be weak relative to CIS under the same measurement conditions. It is therefore unlikely to distinguish CIS and NaInS_2_ using Raman in a phase mixture, unless it contains a very large NaInS_2_ volume fraction.

To confirm the phase identification based on Raman measurements, off‐resonant Raman spectra for different Cu‐In‐S phases were simulated. The computed results for CH‐CIS phase of CuInS_2_, thiospinel structures of *β*‐In_2_S_3_ (space group *I4_1_/amd*)^[^ ^63^ ^]^ and CuIn_5_S_8_ (space group *F* $\bar{4}$ *3m*),^[^ ^64^ ^]^ and delafossite NaInS_2_ (space group *R‐3m*)^[^ ^50^ ^]^ are presented alongside the measured ones in Figure 6b. Clearly, all simulated spectra agree well with their experimental analogues, validating the assignment used in the previous section and proving that the employed simulation methodology is indeed suitable for the material system of interest. Still, a small but systematic shift of all modes is observed for CuIn_5_S_8_, which could be because the partial cationic disorder seen experimentally^[^ ^64^ ^]^ is not captured by our simulations. The key difference though is the absence of the experimental peak at 341 cm^−1^ in the simulated Raman spectra of NaInS_2_. The computed spectrum is consistent with the measurements presented in ref. [73], suggesting that our sample might contain traces of yet another secondary phase. Based on the precursor film composition ([Na]/[In] ≈ 1.5), this phase is expected to be Na‐rich. To verify this hypothesis, additional calculations were performed for Na_3_InS_3_ (space group *C2/c*)^[^ ^65^ ^]^ and Na_5_InS_4_ (space group *P2_1_/m*),^[^ ^66^ ^]^ as shown in Figure S2b, Supporting Information. The obtained results show that the extra peak in the Raman spectra for NaInS_2_ reference sample likely originates from Na_3_InS_3_.

### 3.3 Simulated Raman Spectra of CuInS_2_ Polymorphs

**Figure 7:**
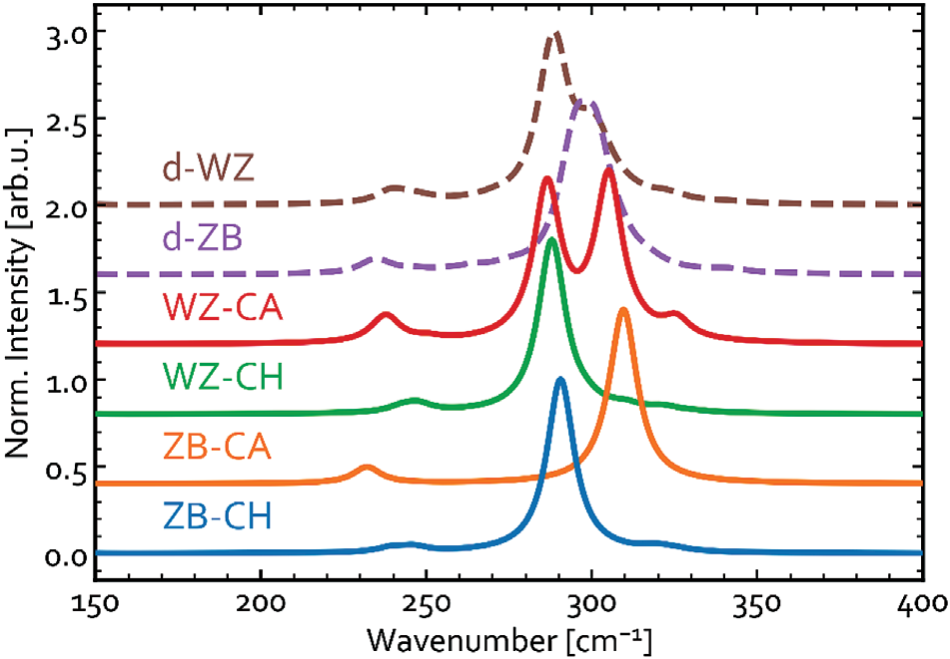
Simulated Raman spectra for the different ordered structures and polytype‐averaged disordered phases of CuInS_2_.

In this section, the simulated Raman spectra for different CuInS_2_ polymorphs introduced earlier are analyzed. The results obtained for the four ordered and two disordered metastable phases are presented in **Figure** **7**. The ordered structures here are modeled explicitly, whereas the disordered ones are represented by Raman spectra averaged for four (for ZB‐derived) or three (for WZ‐derived) different polytypes with 50% CuAu‐type fraction. The individual Raman spectra for these and several other polytypes with different CA‐type fractions are shown in Figure S8, Supporting Information. As one can see, all six CIS phases have the dominant signals at the wavenumbers between 280 and 320 cm^−1^. As expected, the position of the dominant peak for ZB‐CH almost perfectly matches the experimental signal at 292 cm^−1^,^[^ ^75^ ^]^ but it unfortunately also overlaps with the dominant peak for WZ‐CH CuInS_2_ and even one intense peak for WZ‐CA. Thus, ZB‐CH and WZ‐CH structures are indiscernible with Raman. It is expected, however, that this peak in most cases is dominated by ZB‐CH, simply because it is the most stable at the conditions of growth and storage. The shoulder at around 305 cm^−1^ in the experimental spectrum, which is typically attributed to ZB‐CA in literature,^[^ ^39^ ^]^ can as well be explained by d‐ZB, d‐WZ, and WZ‐CA as all these yield similar signals in the simulated spectra.

### 3.4 Properties of Cu‐In‐S Precursors

**Figure 8:**
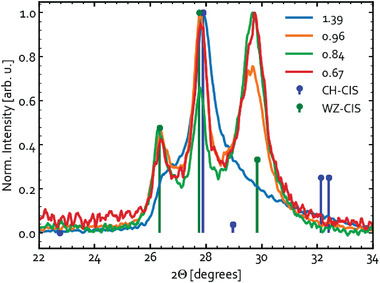
GIXRD pattern of precursors with different [Cu]/[In] ratios expressed in the legend.

While the previous sections focused on the results of calculations and measured Raman spectra of reference materials, this section describes experimental work on sputtered CIS precursors with different compositions. The precursors sputtered without intentional substrate heating are characterized by Raman and GIXRD to understand their phase compositions. **Figure** **8** shows the diffractograms measured for four precursors with compositions varying from Cu‐poor ([Cu]/[In] = 0.67) to Cu‐rich ([Cu]/[In] = 1.39). The figure shows only a narrow range of 2*θ* angles for convenience of phase identification. The corresponding full‐range XRD patterns are available in Figure S9, Supporting Information.

The fact that clear peaks are present in the diffractograms indicates that the deposits partially crystallize during sputtering even without intentional substrate heating. A significant fraction of the layer may remain amorphous though. The reflections of the reference from the Inorganic Crystal Structure Database (ICSD)^[^ ^91^ ^]^ for the thermodynamically stable CH‐CIS phase (collection #66 865^[^ ^67^ ^]^) are indicated with the blue markers in the figure. The diffractogram for Cu‐rich precursor ([Cu]/[In] = 1.39) is found to be dominated by the reflections attributed to the CH‐CIS, CA‐CIS, and/or SPH‐CIS phase. For the near‐stoichiometric ([Cu]/[In] = 0.96) and Cu‐poor ([Cu]/[In] = 0.84 and 0.67) precursors, however, the thermodynamically stable chalcopyrite is clearly not the primary phase formed. Instead, a significant contribution from WZ‐CIS is observed, as indicated with green markers in Figure 8. The reference for the disordered CuInS_2_ wurtzite structure (space group *P6* *_3_* *mc*) is the collection #163 489 in the ICSD database.^[^ ^68^ ^]^ The wurtzite phase has previously only been observed in CIS nanocrystals prepared by low‐temperature solution‐based processing^[^ ^35^, ^36^, ^42^, ^68^, ^80^, ^81^ ^]^ and in electrodeposited films.^[^ ^32^ ^]^ This is the first time the metastable phase has been found in sputtered CuInS_2_ thin films, and it can be justified by the highly non‐equilibrium growth process coupled with the low temperature causing the material to be trapped in the metastable phase. The observation that the WZ‐CIS phase is more prominent in the Cu‐poor precursors may also indicate that wurtzite CIS is more tolerant to off‐stoichiometry.

**Figure 9:**
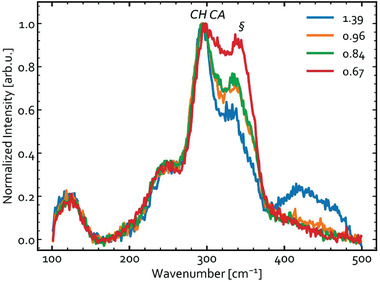
Raman spectra measured with an excitation wavelength of 633 nm for the precursors with various chemical compositions expressed as [Cu]/[In] ratios in the legend.

**Figure** **9** shows the Raman spectra of the precursors measured with an excitation wavelength of 633 nm. The spectra obtained with 532 and 785 nm excitation are shown in Figures S14 and S15, Supporting Information. The Raman spectra are dominated by relatively broad features, indicating small domain sizes. This is not surprising given the low processing temperature. In literature the main A_1_ mode of CH‐CIS is typically observed at around 292 cm^−1^,^[^ ^75^ ^]^ while the A_1_ mode of CA‐CIS appears at 305 cm^−1^.^[^ ^39^ ^]^ It should, however, be kept in mind that the results of our calculations indicate that d‐ZB, d‐WZ, WZ‐CA, and ZB‐CA structures all have a peak near 305 cm^−1^. The broad nature of the dominant peak could therefore include overlapping contributions from several polymorphs.

The main difference in the Raman spectra in Figure 9 relates to the mode labeled “§” centered around 340 cm^−1^. This mode is clearly increased in intensity for samples with lower Cu content. The assignment of this peak is, however, not straightforward. The 340 cm^−1^ vibration could coincide with E(L)/B_2_(L) in CH‐CIS at 345 cm^−1^.^[^ ^43^ ^]^ The E(L)/B_2_(L) typically does not show up in the spectrum of CH‐CIS when using 532 or 633 nm excitation.^[^ ^82^ ^]^ This mode can, however, be observed under resonant Raman conditions due to a break‐down of the Raman selection rule as reported previously.^[^ ^75^ ^]^ In our samples, a strong E(L)/B_2_(L) mode is likewise observed when using 785 nm, which is a near‐resonance conditions for CIS (see Figure S15, Supporting Information). The fact that a 340 cm^−1^ mode also shows up under non‐resonant conditions (633 nm excitation wavelength) indicates that either near‐resonance is in fact playing a role or that this peak could relate to a different phase or compound than CH‐CIS. The two candidates here are Na_3_InS_3_ and CuIn_5_S_8_ (see Figure 6 and Figure S2, Supporting Information). CuIn_5_S_8_ is not found by XRD in the precursor, while the presence of Na_3_InS_3_ would be surprising since a large Na surplus is required to form this compound. As demonstrated in the next section and in Figure S24, Supporting Information, a correlation exists between the quantity of WZ‐CIS phase extracted from XRD and the 340 cm^−1^ peak intensity in Raman with 633 nm excitation. The peak cannot, however, be assigned to any CIS polymorph under non‐resonant conditions, as none of these have a strong signal around 340 cm^−1^ in the simulation (see Figure 7). A peak at 340 cm^−1^ was previously recorded for CuInS_2_ nanoparticles^[^ ^41^, ^83^ ^]^ and attributed to WZ‐CA based on the analysis of computed vibration frequencies,^[^ ^41^ ^]^ but our results reveal that this mode is either very weak or silent under non‐resonant conditions, suggesting that the signal could belong to hitherto unidentified phase or structure. All our characterization results seem to imply that this phase has wurtzite lattice and [Cu]/[In] considerably below unity, which might be the stabilizing factor. An attempt was made to identify this structure by using high‐throughput screening of CuInSe_2_‐In_2_Se_3_ pseudo‐binary system, but all discovered WZ structures have energies high above the convex hull, even higher than the unstable ZB‐derived ordered vacancy compounds of the same composition, see Figure S25, Supporting Information. Hence, the origin of the Raman signal at 340 cm^−1^ remains to be determined.

The Raman spectra obtained using 532 nm excitation (see Figure S14, Supporting Information) furthermore suggests that even the precursors with [Cu]/[In] = 0.84 contains inclusions of Cu*_x_* S, as evidenced by a peak at 475 cm^−1^. The fact that this phase is observed in Cu‐poor precursors further proves that the precursors are far from thermodynamic equilibrium.

### 3.5 Annealed Absorbers

#### 3.5.1 Microstructure

**Figure 10:**
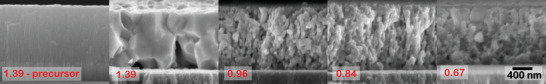
SEM cross‐section of a Cu‐rich precursor and absorbers annealed at 600 °C for 30 min. The numbers in the images refers to the [Cu]/[In] ratios. The same scale bar applies to all images.

**Figure**  **10** shows SEM cross‐sections of samples annealed at 600 °C for 30 min along with the Cu‐rich precursor. It is observed that the composition of the precursors has a strong impact on the resulting microstructure after annealing. For the annealed Cu‐rich sample ([Cu]/[In] = 1.39), large grains extending though the entire film thickness are formed. For all the Cu‐poor samples, on the contrary, the layer consists of very fine grains. The most Cu‐poor film stands out since a few larger grains are found at the surface. These are likely to be CuIn_5_S_8_ particles as demonstrated later. The observed microstructure is similar to co‐evaporated CIS, where small grains are seen in the material grown under Cu‐poor deposition conditions and large grains form when growing Cu‐rich absorbers.^[^ ^84^ ^]^ In order to determine the phase composition of the fine‐grained layers, XRD and Raman measurements were performed on a series of samples annealed at different temperatures. An additional sample with 20 nm NaF deposited on the precursor surface prior to annealing was prepared and characterized to evaluate the impact of Na on the phase formation.

#### 3.5.2 Analysis of Co‐Existing Phases

**Figure 11:**
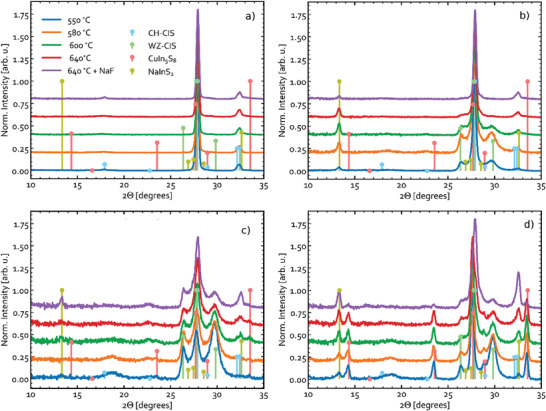
GIXRD (*d*_inc_ = 1°) patterns of Cu‐In‐S samples after annealing at various temperatures. The [Cu]/[In] ratios of the samples are a) 1.39, b) 0.96, c) 0.84, and d) 0.67. The reference patterns in this figure are CH‐CIS (#66 865),^[^ ^67^ ^]^ WZ‐CIS (#163 489),^[^ ^68^ ^]^ CuIn_5_S_8_ (#16 423),^[^ ^64^ ^]^ and NaInS_2_ (#640 036),^[^ ^50^ ^]^ as taken from the ICSD database. The same legend applies to all subfigures.

**Figure** **11** shows a magnified view of the XRD patterns measured for the annealed Cu‐In‐S samples. This range contains signatures of the relevant phases. The complete XRD patterns are available in Figures S10–S13, Supporting Information. As seen in Figure 11a, the Cu‐rich samples do not contain significant amounts of WZ‐CIS after annealing irrespective of the temperature. The small WZ‐CIS contribution seen in the precursor is gone after annealing, and the pattern is now dominated by CH‐CIS, as evidenced by the peak at 2*θ* ≈ 17.9°. The presence of Cu*_x_* S, which could be expected at such Cu‐rich compositions, was not detected by XRD (see Figure S10, Supporting Information), presumably due to poor crystallinity. A signal corresponding to Cu*_x_* S phase was instead recorded by Raman spectroscopy (see Figure S16, Supporting Information). For the Cu‐rich samples, the main impact of annealing temperature is sharpening of the XRD reflections due to the promoted grain growth at higher temperatures. In the Cu‐poor samples, the co‐existence of CH‐CIS, CA‐CIS, or SPH‐CIS and WZ‐CIS polymorphs persists after annealing. The near‐stoichiometric sample ([Cu]/[In] = 0.96) in Figure 11b contains a significant WZ‐CIS fraction after annealing at the lowest temperatures, but its amount is gradually reduced with temperature. For the precursor with an additional NaF layer, the WZ‐CIS phase is almost vanished after annealing, and the XRD pattern becomes similar to that of the Cu‐rich sample. The unique peaks of CH‐CIS are however not clearly visible, making it impossible to rule out that the dominating phase is SPH‐CIS. In the more Cu‐poor sample ([Cu]/[In] = 0.84), the WZ‐CIS phase fraction is also reduced with increasing annealing temperature, but a substantial quantity remains even at the highest annealing temperature (see Figure 11c).

Surprisingly, the sample with [Cu]/[In] = 0.84 (see Figure 11c) does not appear to contain CuIn_5_S_8_ phase. Based on the Cu_2_S‐In_2_S_3_ phase diagram, a volume fraction of 9% CuIn_5_S_8_ is expected for this composition. The excess In is instead incorporated into NaInS_2_, as evidenced by the XRD peaks at 13.2° and 32.4° that emerged for all Cu‐poor samples (see Figure 11b–d). This is despite no NaF layer was deposited and thus Na must have in‐diffused form the glass substrate. Therefore, it can be concluded that, provided sufficient Na is available, NaInS_2_ is more likely than CuIn_5_S_8_ to form and accommodate excess In not incorporated into CuInS_2_.

In the most Cu‐poor sample with [Cu]/[In] = 0.67 (see Figure 11d), the CuIn_5_S_8_ phase is clearly observed after annealing. A volume fraction of about 28% CuIn_5_S_8_ is expected if the CH‐CIS and CuIn_5_S_8_ phases were both stoichiometric and in thermodynamic equilibrium. The quantity of CuIn_5_S_8_ may, however, be reduced due to formation of NaInS_2_, similarly to the sample with [Cu]/[In] = 0.84. This trend not only confirms the active role of Na for crystallization of CIS absorbers, but also highlights the competition between different phases for the excess In.

**Figure 12:**
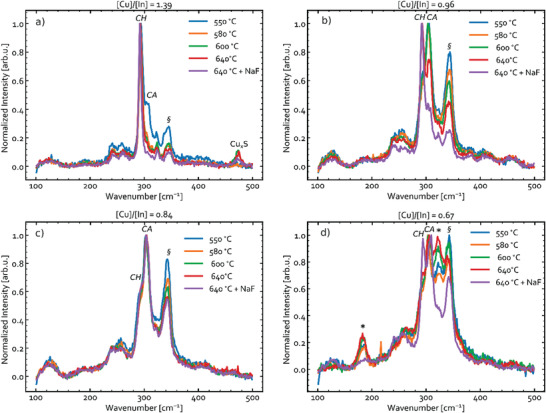
Raman spectra measured with an excitation wavelength *λ*_exc_ = 633 nm for the annealed Cu‐In‐S thin films with the [Cu]/[In] ratios of a) 1.39, b) 0.96, c) 0.84, and d) 0.67. The main contributions from the CIS polymorphs are labeled with “CH” and “CA”. The “*” symbol marks the peaks attributed to CuIn_5_S_8,_ while “§” indicates the ambiguous mode at 340 cm^−1^_._

**Figure** **12** shows Raman spectra measured for the annealed samples with an excitation wavelength of 633 nm. The corresponding spectra obtained using 532 and 785 nm excitation are available in Figures S16–S23, Supporting Information. The Cu‐rich sample (see Figure 12a) has a noticeable contribution from Cu*_x_* S at 475 cm^−1^. This mode is amplified under the 532 nm laser, as seen in Figure S16, Supporting Information. When the samples are annealed at the lowest temperature of 550 °C, a contribution of CA‐CIS at 305 cm^−1^ is visible in the spectrum and the peak at 340 cm^−1^ (marked “§”) is prominent. For samples annealed at higher temperatures, these contributions are reduced and the spectrum is dominated by the A_1_ mode of CH‐CIS at 292 cm^−1^. Here it must be kept in mind that the mode labeled “CH*”* could also relate to other CIS structures like WZ‐CH or WZ‐CA, as shown above (see Figure 7). In the Cu‐poor samples (see Figure 12b–d), the CA‐CIS mode at 305 cm^−1^ is the most prominent. Although CA‐CIS has been claimed observable by XRD, it was not possible to ascribe any reflections to this phase in our samples. The presence of CA‐CIS is therefore only supported by the Raman measurements, and may be questioned in light of the predicted peak overlap with other CIS structures. For the samples with [Cu]/[In] = 0.96, the CA‐CIS signal dwindle compared to the CH‐CIS A_1_ mode with annealing temperature and addition of NaF. Curiously, this is not the case for the sample with [Cu]/[In] = 0.84. At the same time, the ambiguous mode at about 340 cm^−1^ diminish with temperature in all samples (see Figure 12).

Raman spectra of the most Cu‐poor samples ([Cu]/[In] = 0.67 in Figure 12d) contain peaks at 186 and 324 cm^−1^. These modes were not recorded for any other sample and are assigned to CuIn_5_S_8_ (see Figure 6). In agreement with XRD, this phase is only evident in the most Cu‐poor sample, and strongly reduced when NaF has been added to the precursors.

## 4 Discussion

The formation of CH‐CIS, WZ‐CIS, CA‐CIS, CuIn_5_S_8_, and NaInS_2_ in different proportions is detected in Cu‐poor CIS samples. All these phases except for CA‐CIS are observed by XRD. CA‐CIS is only detected by Raman spectroscopy, but the assignment is ambiguous because the characteristic vibrations may relate to ZB‐CA, WZ‐CA or disordered CIS phases (see Figure 7). NaInS_2_ is only observed in XRD (see Figure 11). Based on the trends in the Raman spectra, it is clear that the CA‐CIS peak (305 cm^−1^) is of a different origin than WZ‐CIS and CuIn_5_S_8._ CA‐CIS is not eliminated upon heating the sample with [Cu]/[In] = 0.84, unlike the 340 cm^−1^ mode that remained unassigned. It is therefore proposed that at least three CIS polymorphs co‐exists in the Cu‐poor CIS: CH‐CIS, WZ‐CIS, and CA‐CIS. It may not be excluded that a disordered phase like SPH‐CIS is present in the samples as well, even though it is unlikely at room temperature, as explained above. The co‐existence of interlaced CH‐ and CA‐ordered CIS domains, as previously observed in nanoparticles,^[^ ^11^ ^]^ might be a more accurate description of the sample morphology at nanoscale. The relative contribution of WZ‐CIS always decreases with increasing temperature, but the Raman peak related to CA‐CIS is more persistent in the Cu‐poor material. This likely stems from the fact that the formation energy of CA‐CIS (which is unstable with respect to CH‐CIS) is much lower than that of WZ‐CIS (see Figure 4).

One can speculate about the causes behind the formation of large fractions of the metastable phases in the Cu‐poor samples. In the more explored selenide Cu‐In‐Se system, Boehnke and Köhn found that SPH phase is stabilized down to room temperature when the composition falls in the range 0.32 < [Cu]/[In] < 0.49 and 1.13 < [Se]/([In]+[Cu]) < 1.20.^[^ ^13^ ^]^ The idea that the metastable CIS polymorphs can be stabilized by off‐stoichiometry is further supported by the phase diagram published by Binsma et al.,^[^ ^12^ ^]^ who found that the WZ‐CIS and SPH‐CIS modifications persist at up to 100 °C lower temperatures when grown with either excess Cu_2_S or excess In_2_S_3_ (see Figure 1). It has furthermore been argued that the disordered nature of WZ‐CIS and SPH‐CIS phases with random distribution of Cu and In on the cation sublattice enhance their flexibility to off‐stoichiometry.^[^ ^34^ ^]^ It seems plausible that the formation of metastable phases is triggered by off‐stoichiometry of the precursors, not only with regard to [Cu]/[In] but also excess S which shifts the composition away from the Cu_2_S‐In_2_S_3_ tie‐line (provided behavior of Cu‐In‐S and Cu‐In‐Se systems are similar). Another potential explanation is a shift in phase balance caused by S deficiency in the films. We consider this scenario unlikely, but it cannot be ruled out since S powder fully evaporated by the end of sulfurization, and hence, saturation of the films with gaseous S might have been incomplete (or even reversed by S loss during the cooldown).

It is more surprising that the metastable WZ‐CIS phase persists after annealing at 640 °C. A previous study on CIS nanoparticles found that WZ‐CIS and CH‐CIS can co‐exist after annealing at temperatures between 200 and 400 °C.^[^ ^37^ ^]^ When annealing the nanoparticles at 500 °C, however, the wurtzite phase completely transformed into CH‐CIS.^[^ ^37^ ^]^ Another study found that WZ‐CIS converted partially into CH‐CIS upon heating to 406 °C, but that heating to 600 °C was necessary to eliminate all traces of this phase.^[^ ^68^ ^]^ It is therefore obvious that WZ‐CIS persists at even higher temperature in this study. This may relate to the overall Cu‐poor compositions of the films, in which the WZ‐CIS precipitates are presumably off‐stoichiometric while CH‐CIS phase is unable to accommodate Cu deficiency. As such, converting Cu‐poor WZ‐CIS into the thermodynamically stable structure would necessitate phase separation into CH‐CIS and CuIn_5_S_8_, which might be slow due to the high reaction barrier.

From the results presented here follows that deposition of high‐quality single‐phase Cu‐poor CuInS_2_ is not a trivial task. It could be accomplished by keeping stoichiometry within the narrow single‐phase region, but the approach of low‐temperature sputtering followed by annealing is particularly unsuited for the undertaking. The situation is exacerbated by the use of binary sputter targets in this work. The complex interplay between the metastable CIS phases in addition to the competition between CuIn_5_S_8_ and NaInS_2_ for the excess In are complicating the matter even further. And once the metastable WZ‐CIS is formed due to the off‐stoichiometry or otherwise, it is extremely difficult to eliminate by post‐deposition annealing. Hence, an alternative deposition strategy with more precise composition control or a different post‐processing route may need to be employed. For instance, sputtering from metal targets would certainly not produce the metastable phases in the first place, which might be the reason why the best Cu(In,Ga)S_2_ solar cell to date was fabricated with the absorber grown by sulfurization of sputtered metallic precursors in H_2_S.^[^ ^8^ ^]^ However, the narrow single‐phase region of CH‐CIS may still cause issues during the subsequent sulfurization.

The adoption of co‐evaporation strategy, which has been particularly successful for selenide Cu(In,Ga)Se_2_, might also be a viable alternative. Since this route ensures sulfur overpressure and the growth process is closer to thermodynamic equilibrium, the formation of metastable phases might be suppressed and their impact on devices alleviated. Indeed, three‐stage co‐evaporation has been recently demonstrated to yield devices with 14–15.5% efficiency.^[^ ^9^, ^85^ ^]^ At the same time, Thomere et al. described an unexpected bilayer morphology of the absorbers stemming from segregation of spinel CuIn_5_S_8_‐like and Cu‐poor tetragonal Cu(In,Ga)S_2_ phases during the second deposition stage.^[^ ^86^ ^]^ The root cause of this behavior is the narrow single‐phase region of CH‐CIS, which is further complicated by the introduction of gallium. On the contrary, Shukla et al. did not report the bi‐layer morphology for films produced via a similar three‐stage co‐evaporation approach,^[^ ^9^ ^]^ indicating that the phase formation is strongly dependent on the deposition parameters and that the described issues can be circumvented, in principle.

## 5 Conclusions

Several metastable CuInS_2_ phases were observed with a series of XRD and Raman measurements on Cu‐poor films produced by room temperature sputtering from binary targets followed by a high‐temperature annealing. Most of these phases have been reported earlier and are already ascribed distinct XRD reflections and/or vibration modes, but our simulations based on the first‐principles calculations reveal that this assignment is ambiguous. Most notably, the A_1_‐S‐S vibration mode at 305 cm^−1^ in Raman spectra of CuInS_2_, which is typically attributed to CA‐CIS, can as well be ascribed to disordered SPH‐CIS or WZ‐CIS, as well as ordered WZ‐CH or WZ‐CA structures. Previously suggested assignment of the Raman peak at about 340 cm^−1^ to WZ‐CIS phase is also shown to lack sufficient evidence. Based on the analysis of computed formation energies for hundreds of CIS polytypes and existing literature, we conclude that CIS grown via non‐equilibrium routes is likely to exhibit complex morphology of interlaced domains with closely‐related ordered structures. Still, the metastable phases with different lattice types could be distinguished by XRD, which allowed us to detect wurtzite CuInS_2_ for the first time in thin films produced by sputtering. While the presence of metastable phases is not particularly surprising in the precursors, the persistence of WZ‐CIS after annealing at 640 °C is unexpected and could pose a practical challenge. The co‐existence and complex interplay between WZ‐CIS, CH‐CIS, CA‐CIS, CuIn_5_S_8_, and NaInS_2_ in the Cu‐poor CIS precludes this approach of producing homogeneous Cu‐poor CIS for solar cell applications. It is argued that the metastable phases are stabilized by off‐stoichiometry at the precursor deposition stage and remain kinetically trapped during post‐deposition annealing. Future attempts to make Cu‐poor CIS must therefore focus on discovering alternative strategies for achieving perfect stoichiometry in order to avoid or effectively eliminate the undesired phases.

## Conflict of Interest

The authors declare no conflict of interest.

## Supporting information

Supporting Information

## Data Availability

The data that support the findings of this study are openly available in Materials Cloud Archive at https://doi.org/10.24435/materialscloud:rb‐ny, reference number 24435.
