# Supplementary material for: Experimental and Theoretical Study of Stable and Metastable Phases in Sputtered CuInS2
Source: Adv Sci (Weinh). 2022 Jun 20;9(23):2200848. doi: 10.1002/advs.202200848 (PMC9376846; doi:10.1002/advs.202200848)
Supplement: Supplementary file 1 — Supporting Information [file ADVS-9-2200848-s001.pdf]

ADVANCED SCIENCE
Open Access

Supporting Information

for *Adv. Sci.*, DOI 10.1002/advs.202200848

Experimental and Theoretical Study of Stable and Metastable Phases in Sputtered $CuInS_2$

Jes K. Larsen, Kostiantyn V. Sopiha*, Clas Persson, Charlotte Platzer-Björkman and Marika Edoff*

Supporting Information for

Experimental and theoretical study of stable and metastable phases in sputtered $CuInS_2$

J. K. Larsen[1,§], K. V. Sopiha[1,§,*], C. Persson[2,3], C. Platzer-Björkman[1], M. Edoff[1]

[1] Division of Solar Cell Technology, Department of Materials Science and Engineering, Uppsala University, Box 534, SE-75237 Uppsala, Sweden

[2] Centre for Materials Science and Nanotechnology/Department of Physics, University of Oslo, Blindern, Box 1048, NO-0316 Oslo, Norway

[3] Department of Materials Science and Engineering, Royal Institute of Technology, SE-10044 Stockholm, Sweden

* Corresponding author: J. K. Larsen (jes.larsen@angstrom.uu.se)

§ These authors contributed equally to the work

1. Reference materials

1.1. $NaInS_2$ reference sample

A $NaInS_2$ reference sample was produced in order to investigate the characteristic Raman peaks of the material. The samples were produced by deposition of 50 nm NaF by e-beam evaporation on soda-lime glass, 30 nm In was deposited onto the NaF. The film was subsequently annealed at 580 °C for 30 min in a graphite reactor with 20 g of elemental sulfur and a 300 mbar Ar background pressure.

The thin film was characterized by GIXRD and Bragg-Brentano XRD to verify that $NaInS_2$ had formed as shown in Figure S 1. Based on the reference pattern for $NaInS_2$ with card #640036 in the ICSD database [1], it is concluded that the $NaInS_2$ phase was successfully produced with this approach.

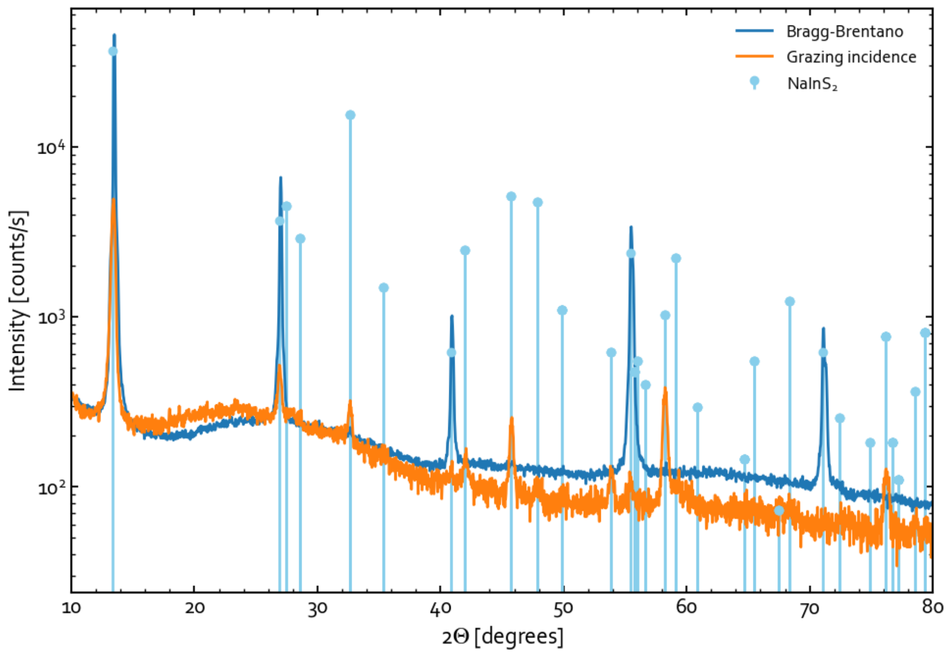

Figure S 1: GIXRD and Bragg-Brentano XRD patterns of a $NaInS_2$ thin film on soda-lime glass. The reference pattern for $NaInS_2$ with card no 640036 from the ICSD database is included to indicate the expected reflections of the compound [1].

Error! Reference source not found. Figure S2 shows the Raman spectra of $NaInS_2$ measured with different excitation wavelengths. Akin to $CuInS_2$, it is noticed that the modes at 258 cm^{-1} and 341 cm^{-1} are enhanced when using the 785 nm laser. This is somewhat surprising since it would not be expected that $NaInS_2$ in resonance with the 785 nm laser. The band gap of $NaInS_2$ has been reported to be around 2.3 eV [2]–[4]. The compound is therefore expected to be in resonance with the 352 nm laser.

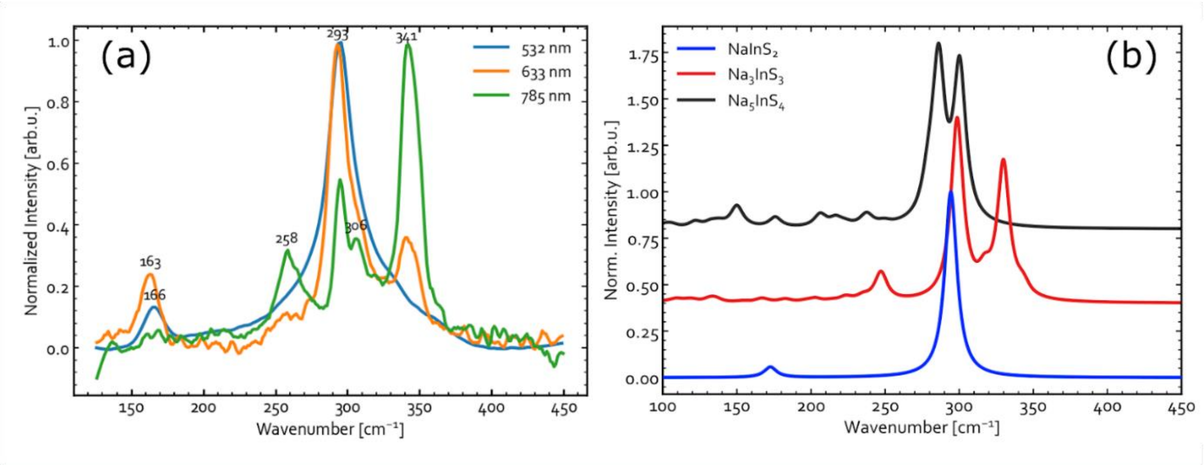

Figure S 2: Raman data for Na-In-S phases. (a) Experimental spectra of $NaInS_2$ reference sample measured using different excitation wavelengths. (b) Simulated spectra of different Na-In-S phases with [Na]/[In]≥1.

1.2. $CuIn_5S_8$ thin film reference

A $CuIn_5S_8$ reference sample was produced to unambiguously verify the Raman spectrum of the compound. Cu and In was deposited on high strain, low Na glass in the ratio [Cu]/[In] = 0.2 by co-evaporation. The precursor was then annealed at 580 °C in a sulfur-containing atmosphere to produce $CuIn_5S_8$. Based on the XRD pattern in reference [5], it can be confirmed that the $CuIn_5S_8$ was formed (see Figure S 3). Since no unidentified peaks are seen in the pattern, it appears that the sample consists of single phase $CuIn_5S_8$. Raman spectra of the sample measured with various excitation wavelengths is available in Figure S 4.

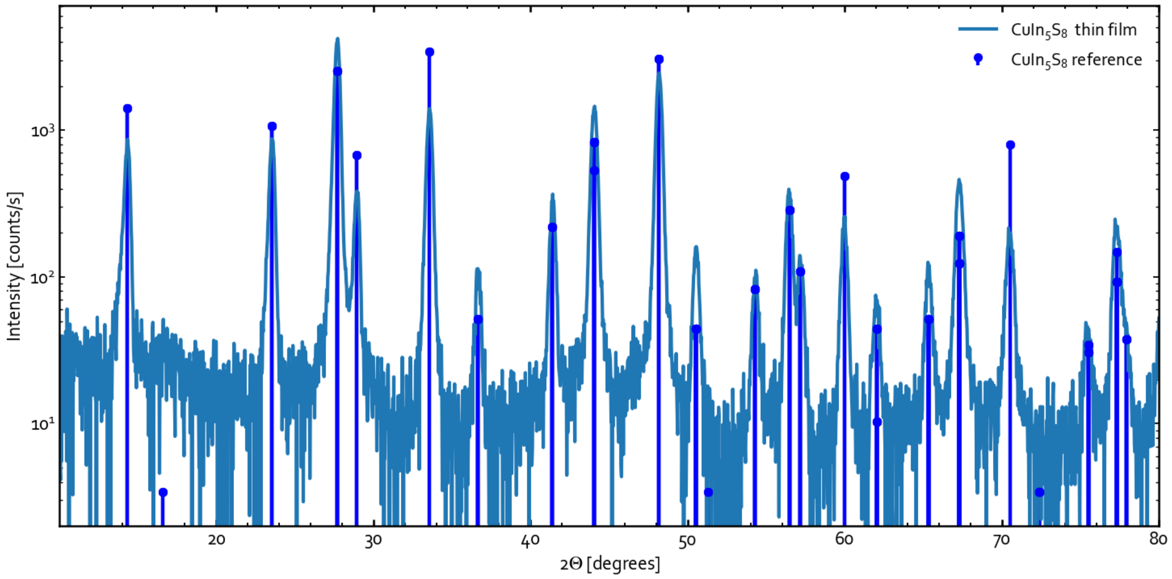

Figure S 3: GIXRD (d_{inc}=1°) of $CuIn_5S_8$ thin film. The reference pattern in is card #16423 in the ICSD, originally published in reference [5].

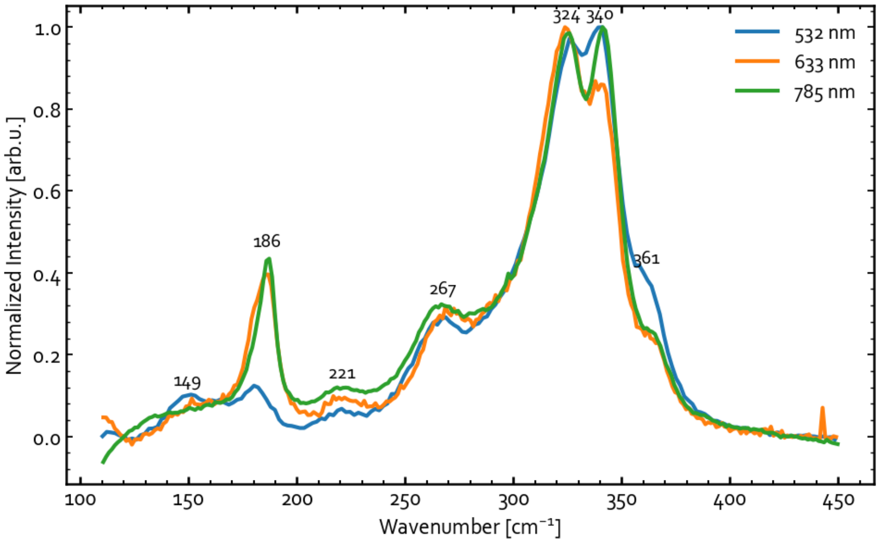

Figure S 4: Raman spectra of $CuIn_5S_8$ measured with various excitation wavelengths indicated in the legend.

1.3. In_2S_3 reference

In_2S_3 was produced by sulfurization of a Indium metal shot at 580 °C. The Raman spectrum measured with 532, 633, and 785 nm excitation is shown in Figure S 5. The characteristic peaks observed in the material are in good agreement with previously published Raman spectra of In_2S_3 [6]–[8].

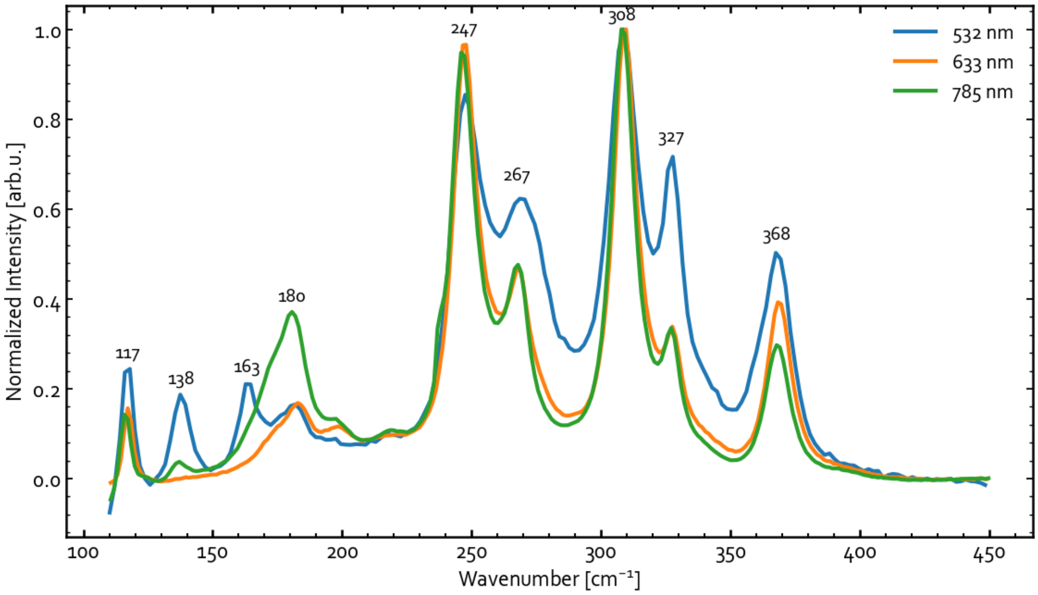

Figure S 5: Raman spectra of In_2S_3 measured with various excitation wavelengths indicated in the legend.

1.4. Comparative Raman spectra

In the main paper the Raman spectra measured with an excitation wavelength of 633 nm were presented. Since the spectra are sensitive to the excitation wavelength we supplement with measurements performed with λ_{exc} = 532, 785 nm in Figure S 6 and Figure S 7.

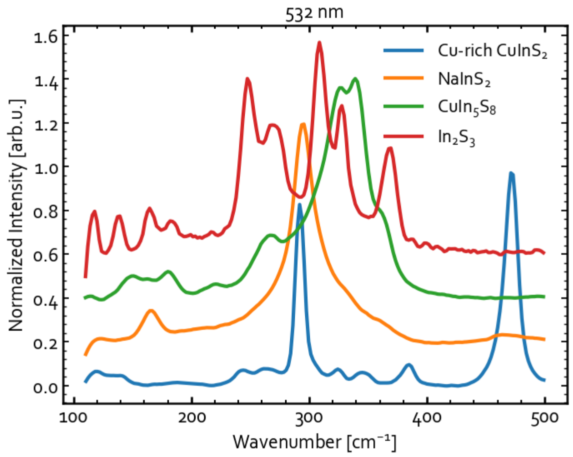

Figure S 6: Raman spectra of reference material measured with λ_{exc} = 532 nm. The peak in the CuInS2 sample at 475 cm^{-1} belongs to Cu_2S.

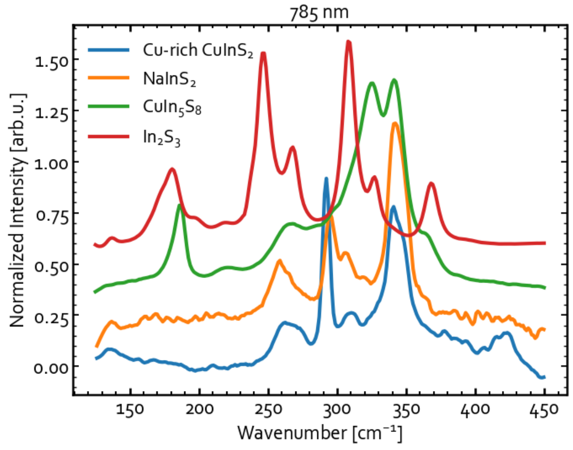

Figure S 7: Raman spectra of reference material measured with λ_{exc} = 785 nm.

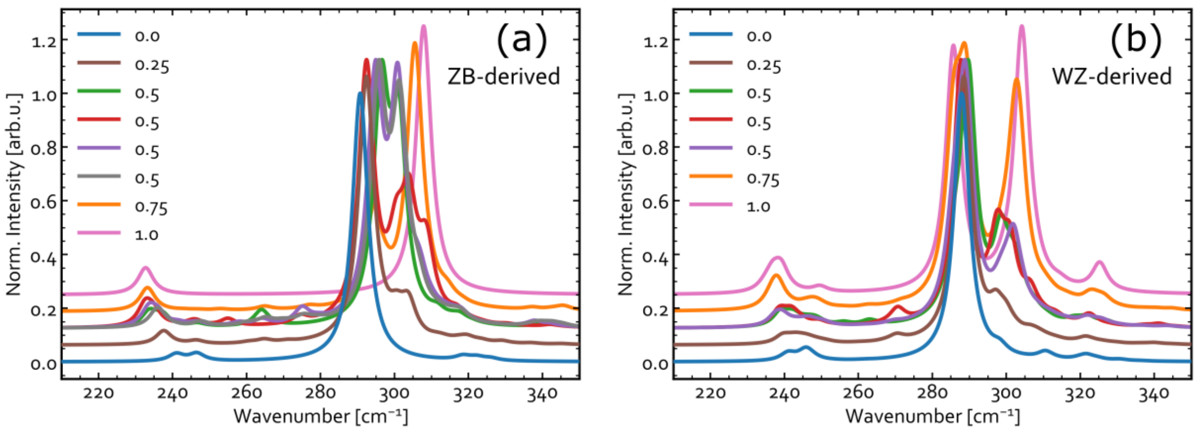

Figure S 8: Simulated Raman spectra of CIS polytypes with (a) ZB-derived and (b) WZ-derived structures. The numbers in legends represent CA-type fractions. To better resolve the peaks, Lorentzian smearing with a smaller FWHM of 5 cm^{-1} is used for these spectra. The red curve in (a) is notably different from the Raman spectra of other CIS polymorphs with CA-type fraction of 0.5 – the outlier is a polytype consisting of two large domains (CH-type and CA-type of 16 atoms each), whereas all other ZB-derived polytypes are composed of multiple small intermixed domains. This result indicates that further increase in the domain size may result in Raman spectra consisting of mixed CH- and CA-type features, but this assumption requires further analysis.

2. XRD patterns with wider ranges

In the main paper a limited 2θ range of the GIXRD measurements was presented in order to more easily distinguish key features. For completeness the full range of the measurements are shown in Figures S9 – S13.

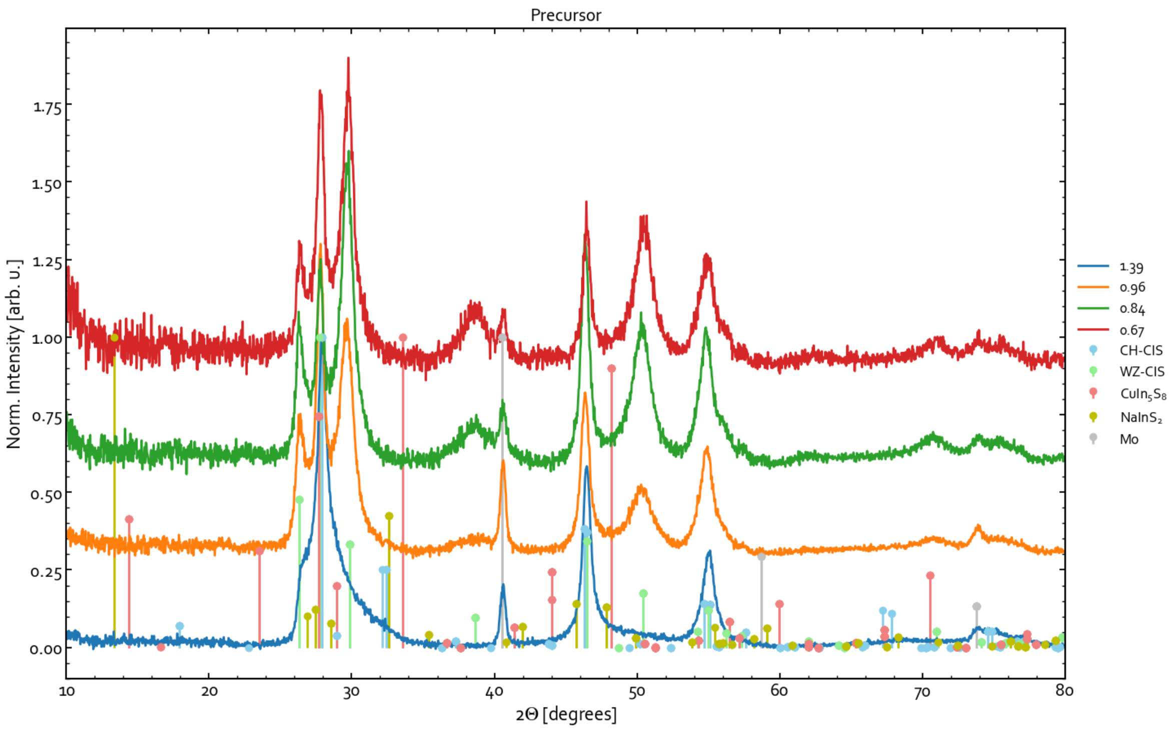

Figure S 9: Complete GIXRD (d_{inc} = 1°) of Cu-In-S precursors with various compositions as indicated by [Cu]/[In] in the legend. The reference patters originate from the ICSD as described in the main paper.

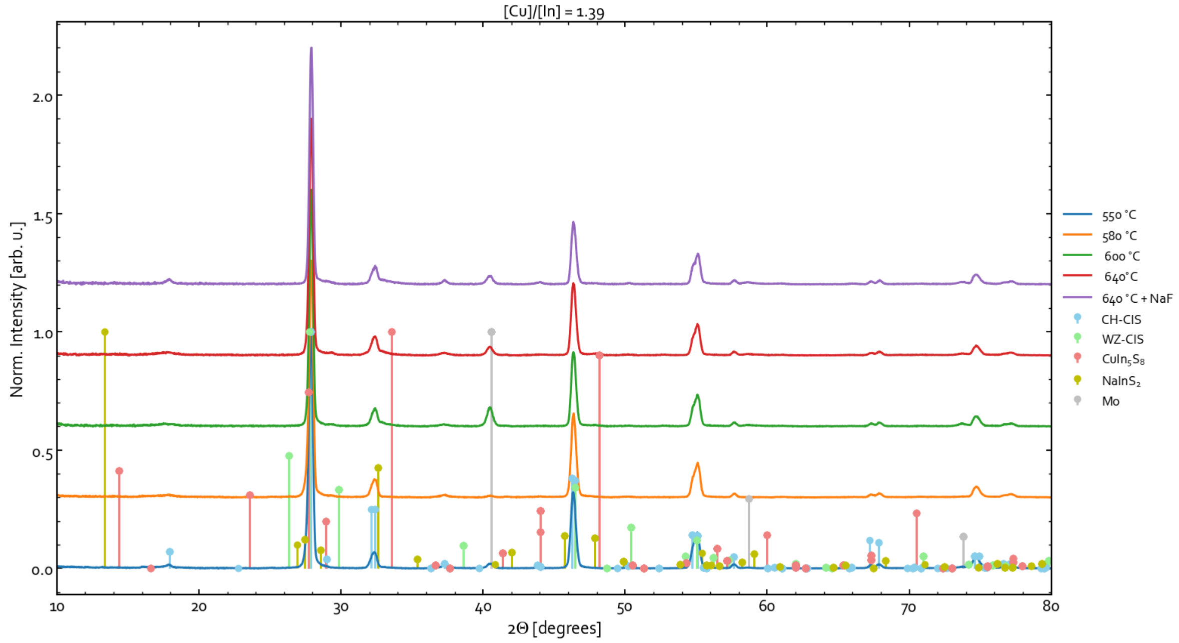

Figure S 10: Complete GIXRD (d_{inc} = 1°) of Cu-In-S samples with [Cu]/[In] = 1.39, annealed at various temperatures. The reference patterns originate from the ICSD as described in the main paper.

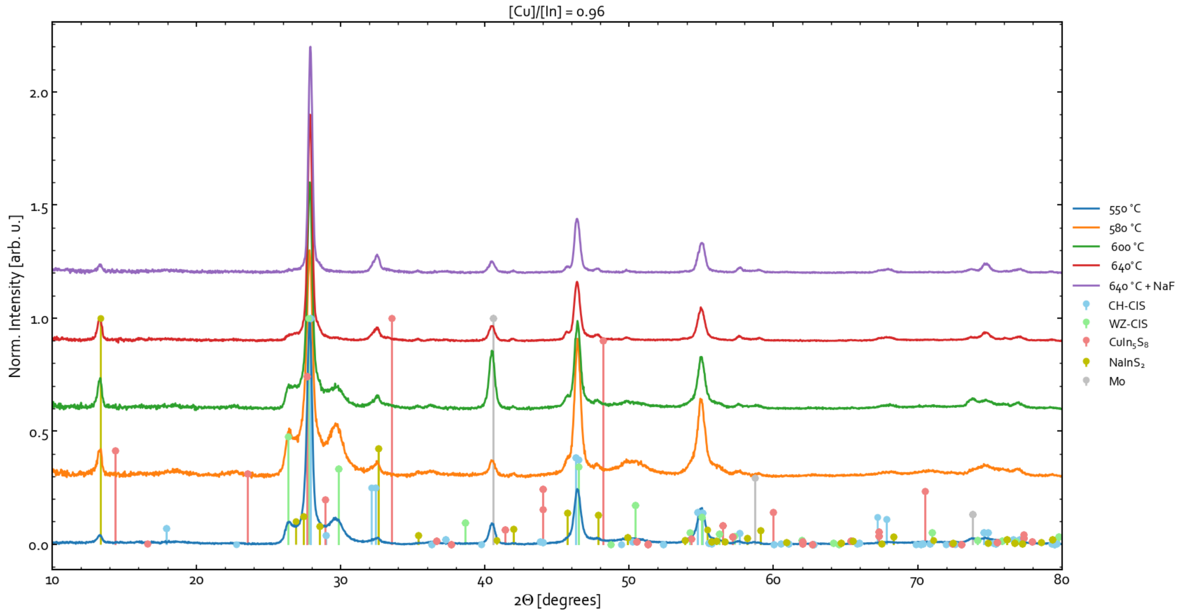

Figure S 11: Complete GIXRD (d_{inc} = 1°) of Cu-In-S samples with [Cu]/[In] = 0.96 annealed at various temperatures. The reference patterns originate from the ICSD as described in the main paper.

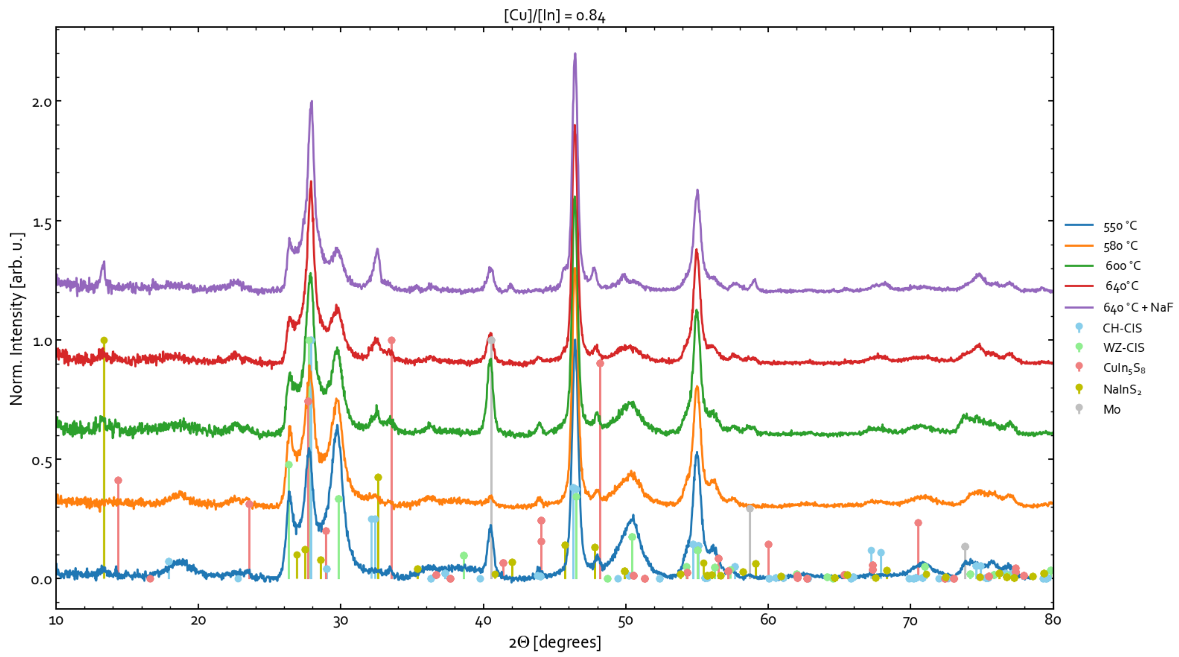

Figure S 12: Complete GIXRD (d_{inc} = 1°) of Cu-In-S samples with [Cu]/[In] = 0.84, annealed at various temperatures. The reference patterns originate from the ICSD as described in the main paper.

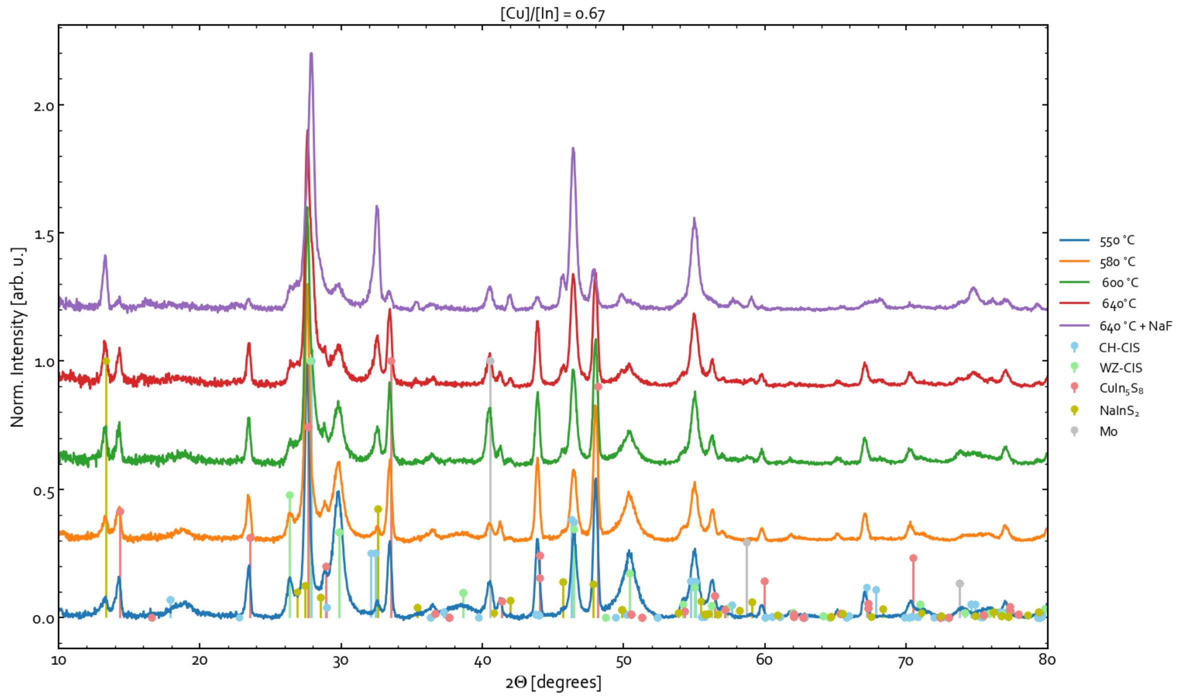

Figure S 13: Complete GIXRD (d_{inc} = 1°) of Cu-In-S sample with [Cu]/[In] = 0.67, annealed at various temperatures. The reference patterns originate from the ICSD as described in the main paper.

3. Raman spectra of all samples

1.5. Precursors

The Raman spectra of the precursors measured with an excitation wavelength of 633 nm were presented in the main paper (Figure 3). For completeness, the in the spectra collected with excitation of 532 and 785 nm lasers are presented in Figure S 14 and Figure S 15.

1.5.1. Excitation wavelength of 532 nm

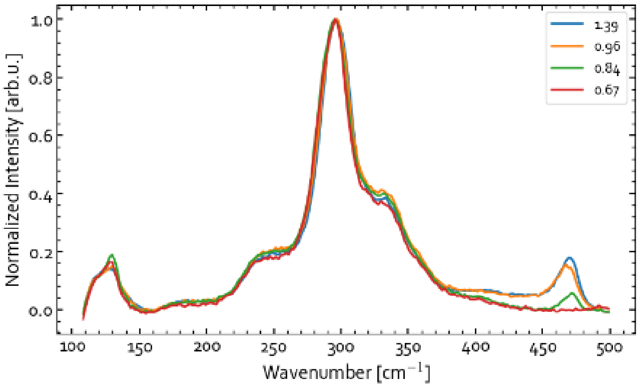

Figure S 14: Raman spectra of Cu-In-S precursors with various compositions expressed as [Cu]/[In] ratios in the legend measured with λ_{exc} = 532 nm.

1.5.2. Excitation wavelength of 785 nm

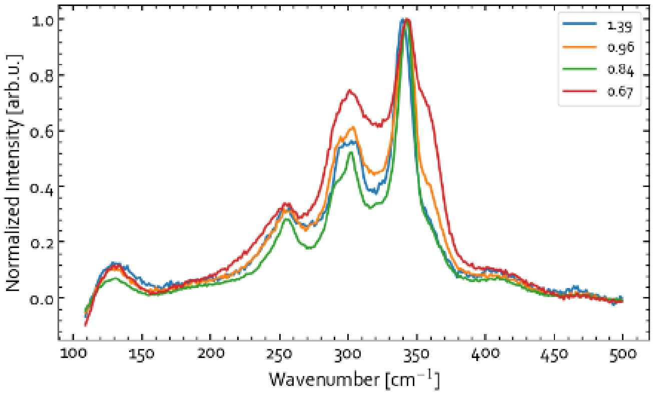

Figure S 15: Raman spectra of Cu-In-S precursors with various compositions expressed as [Cu]/[In] ratios in the legend measured with λ_{exc} = 785 nm.

1.6. Raman of annealed samples

The Raman spectra of the samples in the main paper were measured with an excitation wavelength of 633 nm (Figure 6). For completeness and to illustrate the impact of the excitation wavelength on the Raman spectrum Figures S16-S23 presents the Raman spectra measured with 532 and 785 nm excitation.

1.6.1. Excitation wavelength of 532 nm

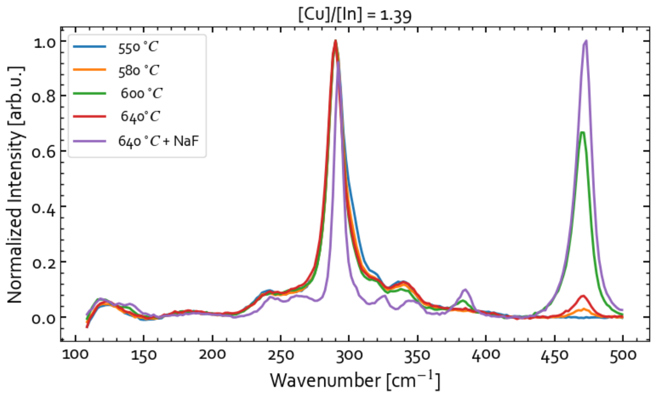

Figure S 16: Raman spectra of CIS with [Cu]/[In] = 1.39 annealed at various temperatures measured with λ_{exc} = 532 nm.

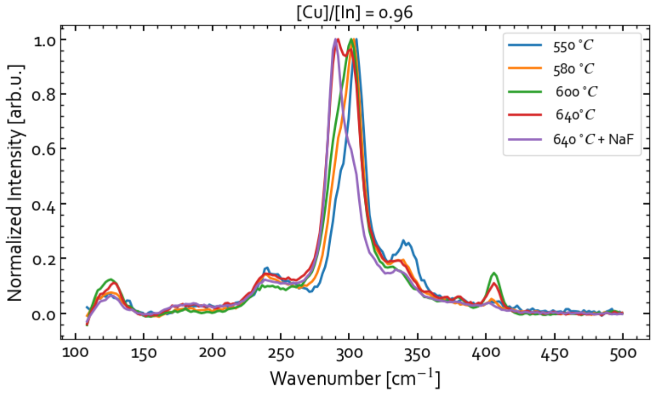

Figure S 17: Raman spectra of CIS with [Cu]/[In] = 0.96 annealed at various temperatures measured with λ_{exc} = 532 nm.

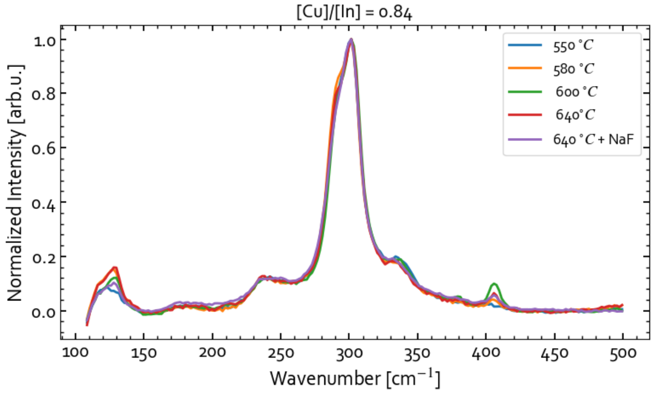

Figure S 18: Raman spectra of CIS with [Cu]/[In] = 0.84 annealed at various temperatures measured with λ_{exc} = 532 nm.

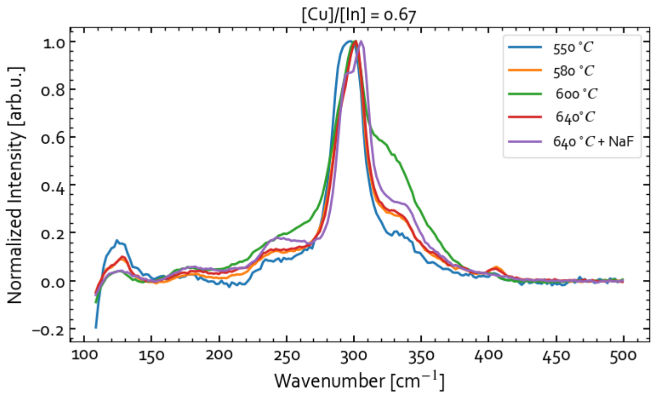

Figure S 19: Raman spectra of CIS with [Cu]/[In] = 0.67 annealed at various temperatures measured with λ_{exc} = 532 nm.

1.6.2. Excitation wavelength of 785 nm

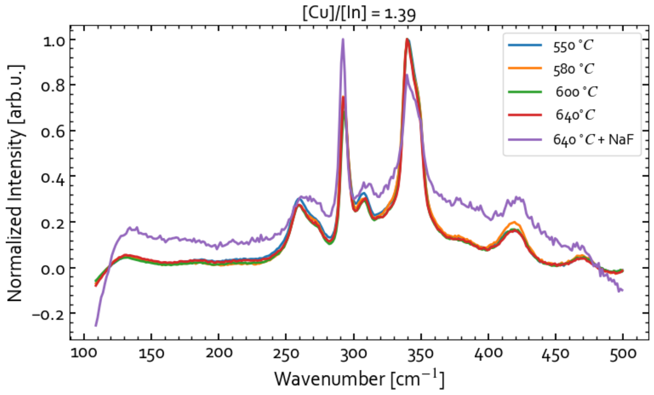

Figure S 20: Raman spectra of CIS with [Cu]/[In] = 1.39 annealed at various temperatures measured with λ_{exc} = 785 nm.

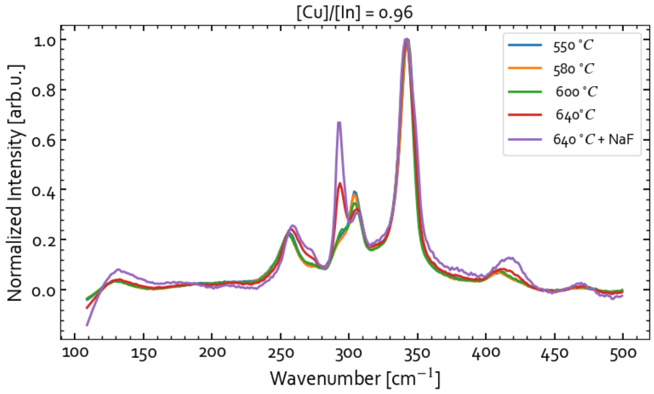

Figure S 21: Raman spectra of CIS with [Cu]/[In] = 0.96 annealed at various temperatures measured with λ_{exc} = 785 nm.

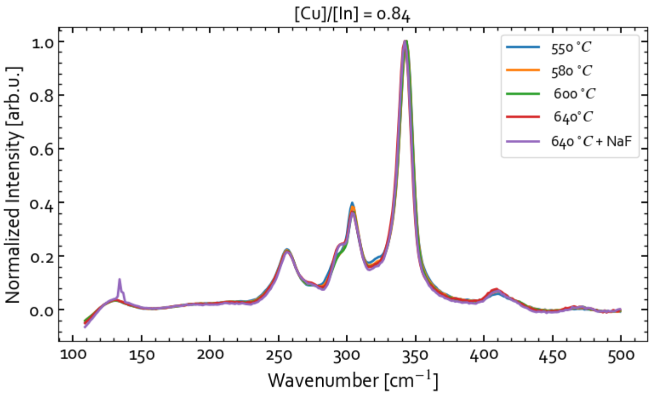

Figure S 22: Raman spectra of CIS with [Cu]/[In] = 0.84 annealed at various temperatures measured with λ_{exc} = 785 nm.

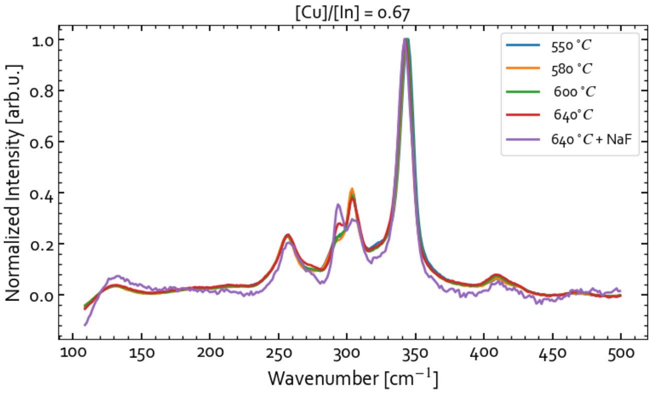

Figure S 23: Raman spectra of CIS with [Cu]/[In] = 0.67 annealed at various temperatures measured with λ_{exc} = 785 nm.

4. Correlation of WZ-CIS phase measured by GIXRD and Raman spectroscopy

Figure S 24 shows the correlation between the peak ratios of XRD and the Raman spectra measured on the same samples. The ratio of XRD peaks ascribed to CH-CIS *(32.3°)* and WZ-CIS *(50.4°)* are plotted versus the ratio of Raman peak intensities at 340 cm^{-1} and the *CH-CIS* A_1 mode at 292 cm^{-1}. The Raman spectra were measured with 633 nm excitation. There is a clear correlation between the WZ-phase measured by XRD and the 340 cm^{-1} peak measured with Raman. (see Figures S20-24).

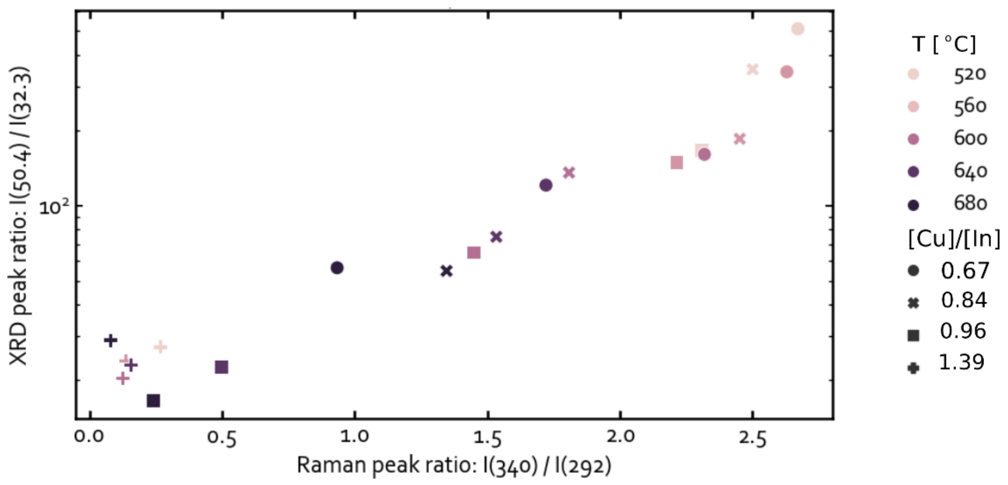

Figure S 24: Correlation of XRD and Raman characteristics. The ratio of XRD peak intensities at I(50.4)/I(32.5) is a measure of the WZ-CIS phase content relative to CH-CIS phase content. The correlation with the Raman peak ratio I(340)/I(292), where I(292) is the A_1 mode of CH-CIS, indicates that the 340 cm^{-1} peak relate to WZ-CIS.

5. Formation enthalpy of Cu-poor Cu-In-S structures

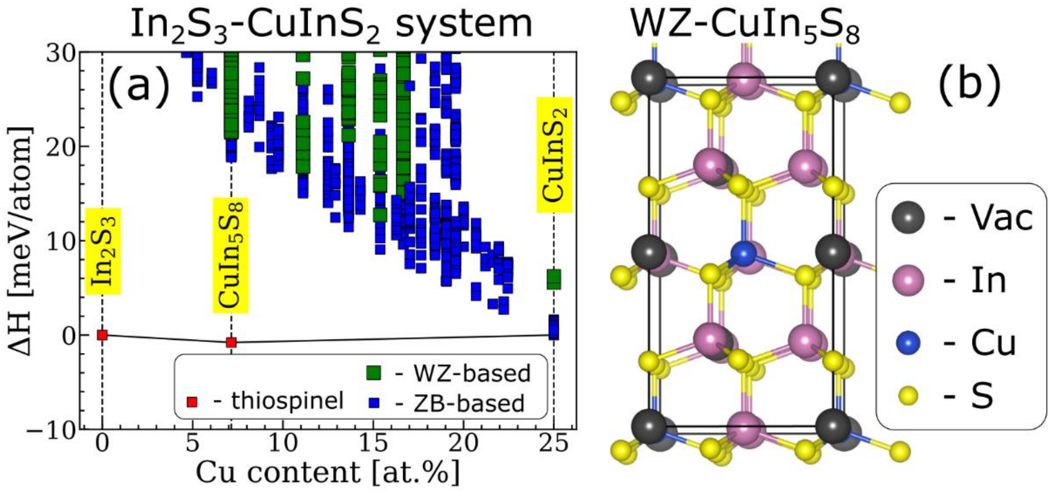

Figure S 25: (a) Convex hull constructed for In_2S_3-$CuInS_2$ pseudo-binary system based on the thiospinel structures of $CuIn_5S_8$ and In_2S_3 from literature, as well as ZB- and WZ-based polytype structures generated using our screening algorithm. (b) The most stable WZ-based $CuIn_5S_8$ structure discovered. Details of the screening algorithm will be published elsewhere shortly.

References:

[1] I. R. Polyvyannyi, V. A. Lata, and V. I. Antonyuk, "Interaction in the Na_2S-In_2S_3 system," *Zh. Neorg. Khim.; (USSR)*, vol. 23:1, Jan. 1978, Accessed: May 12, 2021. [Online]. Available: https://www.osti.gov/etdeweb/biblio/5741408

[2] N. Takahashi *et al.*, "Synthesis, crystal structure and optical absorption of NaInS2-xSex," *Journal of Alloys and Compounds*, vol. 750, pp. 409–413, Jun. 2018, doi: 10.1016/j.jallcom.2018.03.407.

[3] A. K. P. Mann, S. Wicker, and S. E. Skrabalak, "Aerosol-Assisted Molten Salt Synthesis of NaInS2 Nanoplates for Use as a New Photoanode Material," *Advanced Materials*, vol. 24, no. 46, pp. 6186–6191, 2012, doi: https://doi.org/10.1002/adma.201202299.

[4] A. Kudo, A. Nagane, I. Tsuji, and H. Kato, "H2 Evolution from Aqueous Potassium Sulfite Solutions under Visible Light Irradiation over a Novel Sulfide Photocatalyst NaInS2 with a Layered Structure," *Chem. Lett.*, vol. 31, no. 9, pp. 882–883, Sep. 2002, doi: 10.1246/cl.2002.882.

[5] L. Gastaldi and L. Scaramuzza, "Single-crystal structure analysis of the spinel copper pentaindium octasulphide," *Acta Cryst B*, vol. 36, no. 11, Art. no. 11, Nov. 1980, doi: 10.1107/S0567740880009880.

[6] J. Alvarez-Garcia *et al.*, "Raman scattering structural evaluation of CuInS2 thin films," *Thin Solid Films*, vol. 387, no. 1, pp. 216–218, May 2001, doi: 10.1016/S0040-6090(00)01714-4.

[7] K. Kambas, J. Spyridelis, and M. Balkanski, "Far Infrared and Raman Optical Study of α- and β-In2S3 Compounds," *physica status solidi (b)*, vol. 105, no. 1, pp. 291–296, 1981, doi: https://doi.org/10.1002/pssb.2221050132.

[8] E. Kärber, K. Otto, A. Katerski, A. Mere, and M. Krunks, "Raman spectroscopic study of In2S3 films prepared by spray pyrolysis," *Materials Science in Semiconductor Processing*, vol. 25, pp. 137–142, Sep. 2014, doi: 10.1016/j.mssp.2013.10.007.

[9] V. Izquierdo-Roca *et al.*, "Assessment of absorber composition and nanocrystalline phases in CuInS2 based photovoltaic technologies by ex-situ/in-situ resonant Raman scattering measurements," *Solar Energy Materials and Solar Cells*, vol. 95, pp. S83–S88, May 2011, doi: 10.1016/j.solmat.2010.11.014.

[10] K. Wakita, H. Hirooka, S. Yasuda, F. Fujita, and N. Yamamoto, "Resonant Raman scattering and luminescence in CuInS2 crystals," *Journal of Applied Physics*, vol. 83, no. 1, pp. 443–447, Jan. 1998, doi: 10.1063/1.366658.
